# HELZ2 Is an IFN Effector Mediating Suppression of Dengue Virus

**DOI:** 10.3389/fmicb.2017.00240

**Published:** 2017-02-20

**Authors:** Dahlene N. Fusco, Henry Pratt, Stephen Kandilas, Scarlett Se Yun Cheon, Wenyu Lin, D. Alex Cronkite, Megha Basavappa, Kate L. Jeffrey, Anthony Anselmo, Ruslan Sadreyev, Clarence Yapp, Xu Shi, John F. O'Sullivan, Robert E. Gerszten, Takuya Tomaru, Satoshi Yoshino, Tetsurou Satoh, Raymond T. Chung

**Affiliations:** ^1^Gastrointestinal Division, Department of Medicine, Massachusetts General HospitalBoston, MA, USA; ^2^Division of Infectious Diseases, Vaccine and Immunotherapy Center, Department of Medicine, Massachusetts General HospitalBoston, MA, USA; ^3^Laboratory for Systems Pharmacology, Harvard Medical SchoolBoston, MA, USA; ^4^Department of Medicine, Athens University Medical SchoolAthens, Greece; ^5^Department of Biology, Wellesley CollegeWellesley, MA, USA; ^6^Department of Molecular Biology, Massachusetts General HospitalBoston, MA, USA; ^7^Division of Cardiology, Department of Medicine, Beth Israel Deaconess Medical CenterBoston, MA, USA; ^8^Division of Cardiology, Department of Medicine, Massachusetts General HospitalBoston, MA, USA; ^9^Department of Medicine and Molecular Science, Gunma University Graduate School of MedicineMaebashi, Japan

**Keywords:** interferon, interferon effector gene (IEG), genes that are required for IFN-mediated suppression of virus

## Abstract

Flaviviral infections including dengue virus are an increasing clinical problem worldwide. Dengue infection triggers host production of the type 1 IFN, IFN alpha, one of the strongest and broadest acting antivirals known. However, dengue virus subverts host IFN signaling at early steps of IFN signal transduction. This subversion allows unbridled viral replication which subsequently triggers ongoing production of IFN which, again, is subverted. Identification of downstream IFN antiviral effectors will provide targets which could be activated to restore broad acting antiviral activity, stopping the signal to produce endogenous IFN at toxic levels. To this end, we performed a targeted functional genomic screen for IFN antiviral effector genes (IEGs), identifying 56 IEGs required for antiviral effects of IFN against fully infectious dengue virus. Dengue IEGs were enriched for genes encoding nuclear receptor interacting proteins, including HELZ2, MAP2K4, SLC27A2, HSP90AA1, and HSP90AB1. We focused on HELZ2 (Helicase With Zinc Finger 2), an IFN stimulated gene and IEG which encodes a promiscuous nuclear factor coactivator that exists in two isoforms. The two unique HELZ2 isoforms are both IFN responsive, contain ISRE elements, and gene products increase in the nucleus upon IFN stimulation. Chromatin immunoprecipitation-sequencing revealed that the HELZ2 complex interacts with triglyceride-regulator LMF1. Mass spectrometry revealed that HELZ2 knockdown cells are depleted of triglyceride subsets. We thus sought to determine whether HELZ2 interacts with a nuclear receptor known to regulate immune response and lipid metabolism, AHR, and identified HELZ2:AHR interactions via co-immunoprecipitation, found that AHR is a dengue IEG, and that an AHR ligand, FICZ, exhibits anti-dengue activity. Primary bone marrow derived macrophages from HELZ2 knockout mice, compared to wild type controls, exhibit enhanced dengue infectivity. Overall, these findings reveal that IFN antiviral response is mediated by HELZ2 transcriptional upregulation, enrichment of HELZ2 protein levels in the nucleus, and activation of a transcriptional program that appears to modulate intracellular lipid state. IEGs identified in this study may serve as both (1) potential targets for host directed antiviral design, downstream of the common flaviviral subversion point, as well as (2) possible biomarkers, whose variation, natural, or iatrogenic, could affect host response to viral infections.

## Introduction

**Figure 1:**
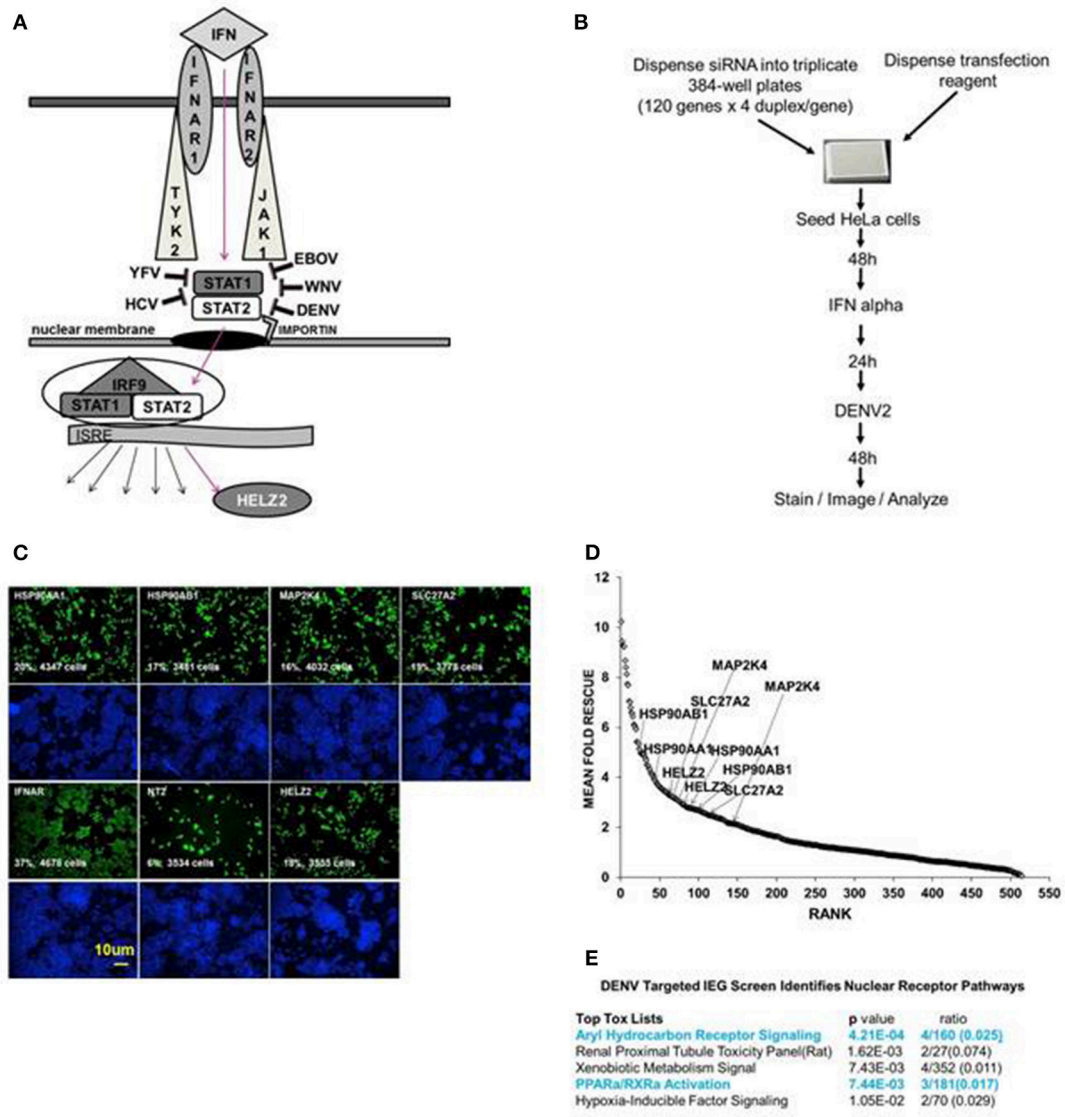
**Targeted Dengue Interferon Effector Screen Identifies 56 DENV-HCV IEGs, including nuclear receptor pathway factors. (A)** IFN signals through IFNAR, JAK1, TYK2, STAT1/2 to activate transcription of IFN stimulated genes, including HELZ2. HELZ2 is a nuclear receptor co-activator and a helicase. **(B)** A targeted RNAi screen was performed to identify host factors required for interferon's antiviral activity against DENV, or DENV IFN effector genes (DENV IEGs). HeLa cells were reverse transfected with 4 unique siRNAs for each of 120 HCV IEGs, followed by 48 h incubation, treatment with IFN, then infection with DENV2, followed by 48 h incubation then staining, imaging, and quantitative analysis. **(C)** Raw Images from DENV IEG screen. HeLa cells were transfected with indicated siRNA, processed as in **(B)**, then stained with anti-DENV antibody (green) or Hoechst nuclear stain (blue) and imaged for quantitative analysis. Cell count and percent infection are indicated on 10x images. **(D)** Rank plot of mean fold rescue for all screened siRNA duplexes. Fold rescue above 1.5 was considered positive. Y axis indicates mean fold rescue, X axis indicates rank of rescue. **(E)** Bioinformatic analysis of DENV IEGs identified two nuclear receptor pathways, the aryl hydrocarbon receptor pathway and the PPARα/RXR pathway.

Adequate therapy is lacking for many clinically relevant flaviviral infections, including dengue virus (DENV), Zika virus (ZIKV), and yellow fever virus (YFV; Acosta and Bartenschlager, 2016; Haug et al., 2016; Nishino et al., 2016). Viral pathology in humans is attributed to both direct consequences of infection and host mediated inflammation (Haug et al., 2016; Kuczera et al., 2016; Valadao et al., 2016). Direct acting antivirals, or therapeutics that specifically inhibit viral replication, have been developed against several viruses [e.g., human immunodeficiency virus (HIV), hepatitis C virus (HCV), hepatitis B virus (HBV)], but are limited by virus-specific activity, development lag time and cost, potential for development of resistance, and lack of effect on pathologic host inflammation. An alternate class of antivirals, host directed antivirals (HDAVs), targets host factors to suppress viral dependency factors or stimulate viral restriction factors, usually modulating a broader antiviral defense system (Acosta and Bartenschlager, 2016). This approach has the benefits of (1) potential activity against many viruses, (2) consolidation of development time/cost, (3) generally lower likelihood of emerging resistance, and (4) potential to restore *effective* host inflammatory response, leading to natural endpoints including negative feedback that will limit excess, ineffective inflammatory stimuli. For these reasons, investigations have focused on exploiting antiviral properties of the host protein interferon alpha (IFN) in a DENV/human cell line model, toward identification of preclinical leads for broad-acting HDAV development. While the antiviral properties of IFN have been known since the 1950s (Isaacs and Lindenmann, 1957), clinical efficacy of IFN is limited due to viral subversion of IFN antiviral effects by many viruses (Figure 1A). By contrast, activation of the IFN pathway downstream of viral subversion might restore host antiviral activity and also limit excess inflammation that is likely triggered by lack of complete IFN signal transduction. To identify the downstream effectors of IFN antiviral activity, we applied a functional genetic approach to DENV because an estimated 3.6 billion of the global population of 7.4 billion people (49%) live in regions at risk for infection, and DENV endemicity is spreading (Wilder-Smith and Gubler, 2008; Louis et al., 2014; Messenger et al., 2014; Butterworth et al., 2016). There is currently no effective treatment for DENV infection, with clinical illness ranging from mild arthralgias to fatal hemorrhagic shock (Horstick et al., 2015). Furthermore, factors predicting likelihood of severe DENV/DENV shock syndrome remain unclear, though repeat infection with antibody dependent enhancement and coinfection with other viruses may contribute to pathogenicity (Halstead, 2015; Perdigao et al., 2016). Efforts to develop an effective vaccine are progressing, but have been challenging (Kirkpatrick et al., 2016; Lourenco and Recker, 2016).

We have previously described a whole genome RNAi screen in which we used fully infectious HCV in human Huh7.5.1 hepatocytes to identify 120 IFN effector genes (IEGs), or genes required for the antiviral effect of IFN against HCV (Fusco et al., 2013). We here tested knockdown of those same 120 HCV IEGs for rescue of fully infectious DENV New Guinea C 2 strain (NGC2) from IFN in HeLa cells, which were selected for tractability (Figure 1B). Building upon our prior studies using a whole genome RNAi screen, here we extend prior work by identifying the functional importance of a novel IFN stimulated helicase and nuclear receptor co activator, HELZ2 (Surapureddi et al., 2002; Tomaru et al., 2006).

## Results

### Screen for DENV IEGs

We aimed to identify human genes required for IFN-mediated suppression of DENV, or DENV interferon effector genes (IEGs). We defined positive hits as those genes for which knockdown led to 1.5-fold or more increase of percent infection, compared to negative controls, for 2 or more unique siRNAs. We found that 56 of the 120 HCV IEGs were also DENV IEGs, required for full antiviral effects of IFN against DENV (Datasheet S1, Figures 1C,D). To further understand how IFN suppresses virus, we then used a bioinformatics approach to look for common pathways among the DENV/HCV IEGs and found enrichment for factors related to nuclear receptors, or ligand-activated transcription factors, including peroxisome proliferator associated protein alpha (PPARα) and aryl hydrocarbon receptor (AHR; Figure 1E). The DENV/HCV IEGs related to nuclear receptors included HELZ2 (Helicase With Zinc Finger 2), a poorly characterized nuclear receptor co-factor (Surapureddi et al., 2002; Tomaru et al., 2006), and nuclear receptor chaperones (HSP90AA1 and HSP90AB1), as well as other host factors described to interact with nuclear receptors (MAP2K4, SLC27A2; Meyer and Perdew, 1999; Bell and Poland, 2000; Hirai et al., 2007; Lebsack et al., 2010; Ahn et al., 2011; Garcia-Fuster et al., 2012; van Steenbeek et al., 2013; Chang et al., 2014; Tsuji et al., 2014; Figures 1C,D). Because HELZ2 is an IEG (required for full IFN antiviral effects), an ISG (upregulated by IFN at the mRNA level), and a helicase, therefore possibly serving as a multi-gene regulator, it seemed likely that HELZ2 was truly required for IFN activity, so we then focused on the mechanism through which HELZ2 mediates IFN antiviral effects (Lanford et al., 2006; Feld et al., 2007; Sarasin-Filipowicz et al., 2008; He et al., 2010; Fusco et al., 2013). Of note, humans express two HELZ2 isoforms (Tomaru et al., 2006), both of which were targeted by the siRNAs used in these assays. No mechanistic difference between isoforms has been described to date.

### Out-of-screen confirmatory studies

**Figure 2:**
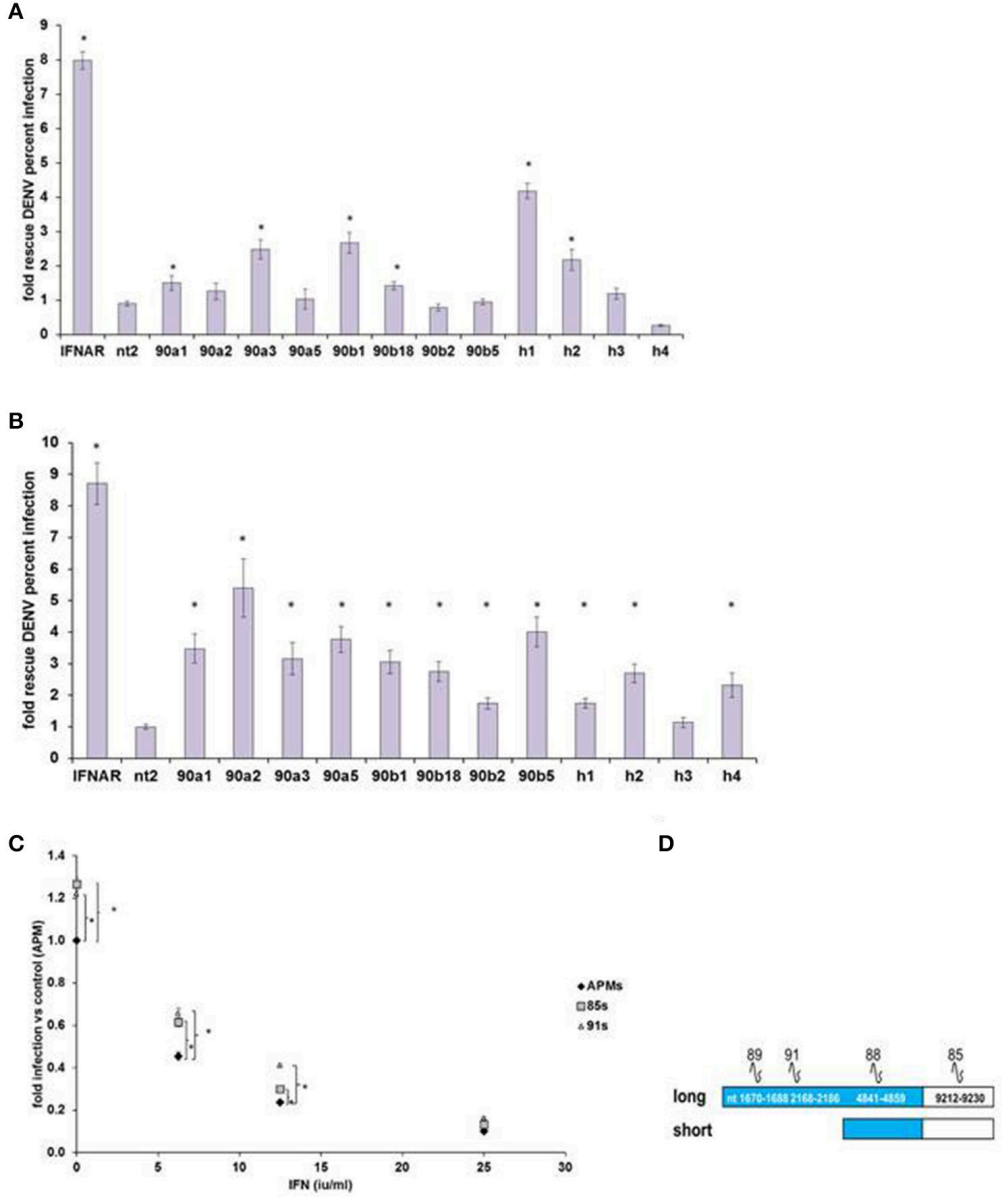
**DENV IEG Screen Hits Confirmation. (A)** HeLa Cell Out-of-Screen DENV IEG Confirmation. RNAi mediated knockdown of select DENV IEG screen hits confirmed rescue of DENV from IFN (HeLa Cells, *n* = 3–5). **(B)** IEG Phenotype Evaluation in a more clinically relevant cell type: hepatocytes. Huh7.5.1 cells were transfected with indicated siRNA followed by treatment with IFN, then infection with DENV, and staining, quantitative imaging. **(C)** Stable HELZ2 knockdown cells exhibit IFN resistance. Huh7.5.1 cells stably transduced with vector control (APM), shRNA targeting HELZ2 long and short isoforms (85 s) or long isoform only (91) were evaluated for IFN resistance. HELZ2 knockdown cells exhibit IFN resistance compared to vector transduced controls. For **(A–C)**, cells were treated with IFN then infected with DENV, followed by staining for DENV and nucleus, then quantitative microscopy for percent infection. Stars indicate significant differences by two-tailed *t*-tests, unequal variance. **(D)** Knockdown shRNA target schematic.

We then confirmed IEG status for screen hits HELZ2, HSP90AA1, and HSP90AB1, verifying that RNAi-mediated knockdown of each target gene led to rescue of DENV from IFN in HeLa cells outside of screening conditions (Figure 2A). We next examined the role of these genes in IFN-mediated DENV suppression in *hepatocytes*, a clinical target cell for DENV infection (Couvelard et al., 1999; Limonta et al., 2007; Aye et al., 2014; Povoa et al., 2014; Figure 2B). RNAi-mediated knockdown of HELZ2 led to DENV rescue from IFN in both HeLa cells and hepatocytes. Furthermore, hepatocytes stably transduced with shRNA against HELZ2, vs. vector control, also displayed reproducible rescue of DENV from IFN, though the DENV-rescue phenotype was more pronounced at lower doses of IFN, compared to HeLa cells (Figures 2C,D).

### Validation of target gene knockdown at mRNA and protein level

**Figure 3:**
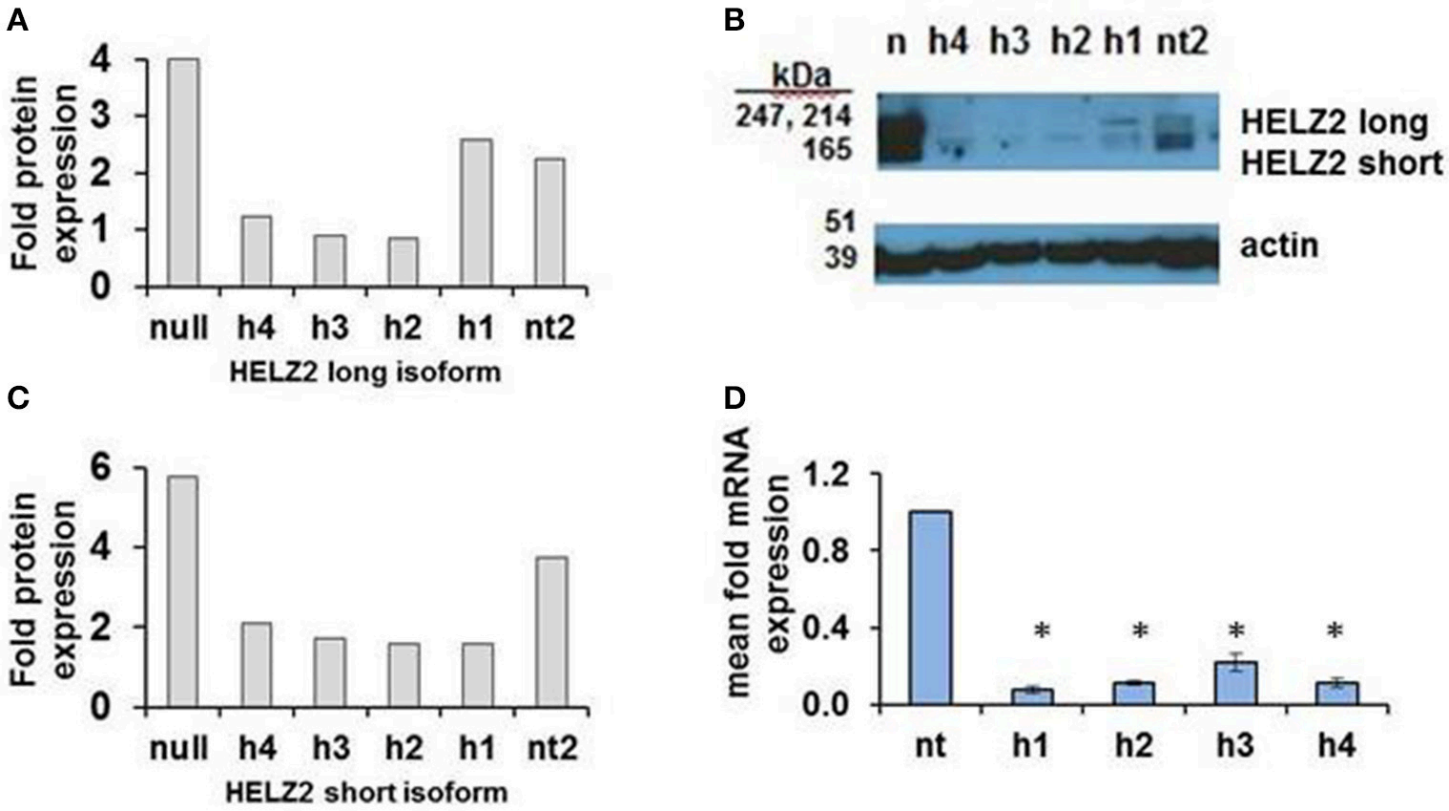
**Validation of HELZ2 knockdown in HeLa cells at the mRNA and protein levels**. HeLa cells were transfected with four unique siRNAs targeting HELZ2 (h1-4), or non-targeting control (nt), followed by 48 h incubation, then IFN treatment for 24 h, then protein or RNA harvest. Quantification of HELZ2 protein, normalized to actin, from a representative western blot is shown for HELZ2 long isoform **(A)**, short isoform **(B)**, with western blot **(C)**. Non transfected (null) sample is indicated by n. qRT PCR was used to validate HELZ2 mRNA knockdown **(D)**. ^*^p < 0.05 compared to control.

HELZ2 was then selected for validation of gene-target knockdown. Validation of long and short isoform protein knockdown by four unique HELZ2 siRNAs was performed using western blot (Figures 3A–C). Validation of mRNA knockdown by four unique siRNAs was performed using qRT PCR (Figure 3D), and revealed ~75% or more fold knockdown of HELZ2 by each unique siRNA duplex.

### Mechanism of HELZ2 IFN effector function

#### Activation

**Figure 4:**
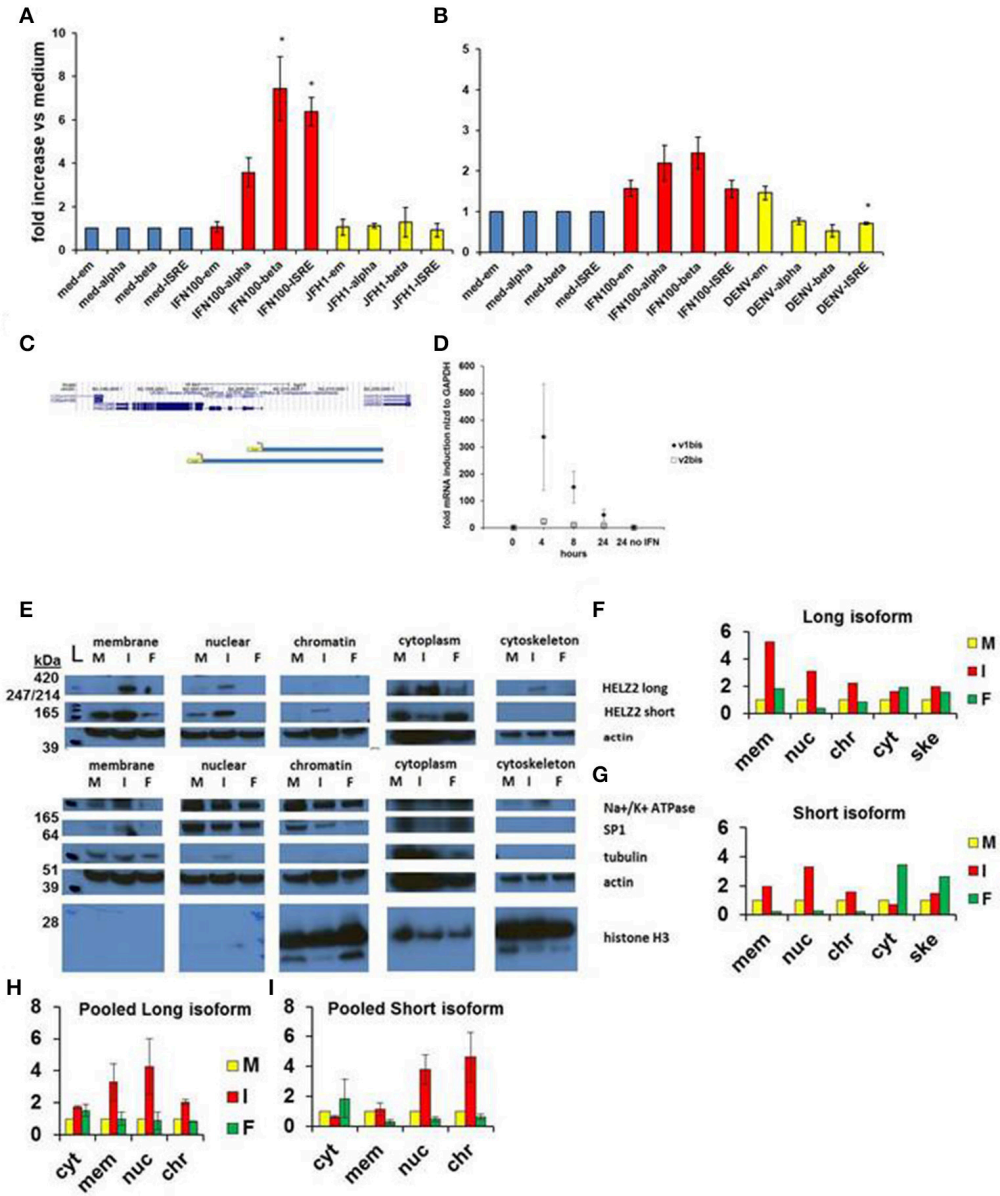
**HELZ2 long (beta, v1) and short (alpha, v2) isoforms are both IFN responsive. (A,B)** HELZ2 alpha, beta promoter activation. Huh751 or HeLa cells were transiently transfected with plasmids containing HELZ2v1 (beta, long isoform) or HELZv2 (alpha, short isoform) promoter with luminescence reporter. Cells were treated with medium, IFN (dose indicated) or virus for 24 h, followed by development and luminescence read. Virus used was JFH1 HCV in Huh751 s and NGC2 DENV in HeLas. **(A)** IFN led to greater HELZ2 v1/beta than v2/alpha promoter activation in Huh7.5.1 human hepatocytes. **(B)** HeLa IFN response is attenuated compared to Huh7.5.1 cells. Virus does not affect promoter response. **(C)** Promoter reporter schematic. The top purple lines indicate two unique HELZ2 promoters, with different transcription start sites for the two HELZ2 isoforms identified based on genomic sequence information from the UCSC genome browser. The lower blue schema represent the two unique luciferase promoter reporters generated for each of the HELZ2 isoform promoters. **(D)** HELZ2 isoform specific mRNA expression. Response of HELZ2 mRNA to IFN time course was measured using isoform-specific primers and qRT PCR. **(E–H)** HELZ2 long (v1/alpha) and short (v2/beta) isoform proteins are upregulated by IFN but not the PPARα agonist fenofibrate. **(E)** Huh7.5.1 cellular compartments were fractionated followed by western blot (wb) for compartment specific HELZ2 expression analyses after 24 h stimulation with medium (M), IFN 1,600 IU/ml (I), or 10 um fenofibrate (F). Experiment was performed thrice and a representative WB is shown **(E)**. HELZ2 long isoform was detected between 420 and 247 kDa bands at highest levels during IFN treatment, with IFN induction in both nuclear and non-nuclear compartments. HELZ2 short isoform protein was also enhanced by IFN, most significantly in the nuclear compartment. **(F,G)** Representative WB results from e. were quantified for each compartment (x axis), with y axis indicating fold increase above background, which was set to 1. mem, membrane; nuc, nucleus; chr, chromatin; cyt, cytoplasm; ske, cytoskeleton. **(H,I)** Pooled quantitative results for three compartment—specific HELZ2 WBs, including **(E)** are shown for HELZ2 long **(H)** and short **(I)** isoforms. Raw data for each pooled experiment is presented in Datasheets S2 and S5.

Based on genomic sequence information from the UCSC genome browser, we identified different transcription start sites for the two isoforms of HELZ2, indicating that the unique HELZ2 isoforms are activated by distinct promoters (Figure 4C). We generated unique luciferase promoter reporters for each of the HELZ2 isoform promoters (Figure 4C), and examined isoform-specific response to IFN stimulation in hepatocytes (Figure 4A, Figures S1A–D), and found that both HELZ2 isoform promoters are activated by IFN, with slightly greater activation of the long vs. short isoform. We repeated these promoter reporter experiments in HeLa cells, and noted much lower IFN activation of both HELZ2 isoforms in HeLa cells compared to hepatocytes (Figure 4B, Figures S1E–H). Search of the HELZ2 promoter sequences revealed the presence of two distinct ISREs in the HELZ2 long (beta) isoform promoter and no ISREs in the HELZ2 short (alpha) isoform promoter (Tsukahara et al., 2006; Datasheets S2 and S5). Of note the four unique ISRE sequences detected in the HELZ2 promoter were slightly different from published ISRE sequences, and might therefore be considered unique ISREs (Tsukahara et al., 2006).

We also attempted to examine the effect of IFN stimulation on isoform-specific mRNA upregulation, using isoform specific HELZ2 primers. Though there was a trend toward the long isoform of HELZ2 being more IFN-responsive than short-isoform HELZ2 (Figure 4D), results were highly variable, limiting interpretability. We next examined the effect of IFN on HELZ2 protein levels and localization, using cellular fractionation followed by western blot. We found that, in hepatocytes, HELZ2-long isoform is detectable only upon IFN stimulation, whereas HELZ2-short isoform is detected in the absence of IFN, but upregulated upon IFN stimulation (Figures 4E–I, Figure S1). Of note, the antibody used in our HELZ2 protein analyses binds an epitope that is present in both HELZ2 isoforms, and is therefore not isoform specific. However, we were able to detect unique bands at roughly 200 and 290 kDa using Western blot, consistent with the long and short HELZ2 isoforms, respectively. In contrast to hepatocytes, HeLa cells displayed detectable HELZ2-short protein, but no detectable HELZ2-long protein, using the abcam anti-HELZ2 Ab (ab129781). In HeLa cells, HELZ2-short protein was not upregulated by IFN (data not shown). This decreased protein expression and lack of protein IFN-responsiveness of HELZ2 in HeLa cells, compared to Huh7.5.1 s, could explain in part the weaker IEG phenotype of HELZ2 in HeLa compared to Huh7.5.1 cells. HeLa cells, have, however, been found to express mRNA for both HELZ2 long and short isoforms (Tomaru et al., 2006), indicating that HeLa vs. hepatocyte (cell to cell) variation in HELZ2 protein expression levels may be at the translational level, or could be due to limitation of the antibody we used.

#### Lifecycle assays

In order to determine the lifecycle stage at which HELZ2 knockdown enhances DENV infection, we then performed a series of lifecycle assays in human hepatocytes, comparing infection in HELZ2 knockdown (85) vs. vector control (APM) cells (Datasheet S3). To assess early infection, we used high sensitivity RNA *in situ* hybridization 90 min following infection, according to published methods (Savidis et al., 2016). In this assay, we found increased infection levels in cells with HELZ2 knockdown (85) vs. vector controls (APM; Figure S2). We tested HELZ2 knockdown effect on DENV RNA levels at a later time point (24 h post infection) using qRT PCR. These experiments revealed enhanced DENV RNA, both in the presence and absence of IFN pretreatment, in HELZ2 knockdown cells vs. vector controls (Figure S3). To complement our immunofluorescence data, we tested effect of HELZ2 knockdown on DENV protein levels using western blot. Western blot experiments revealed an enrichment of DENV protein in HELZ2 knockdown cells, compared to vector controls (Figure S4). Finally, to determine the effect of HELZ2 knockdown on viral release into supernatant, we performed supernatant plaque assays. While these results exhibited significant variability, for each experiment (*n* = 3) supernatant from HELZ2 knockdown cells generated more plaques than supernatant from vector controls (Figure S5). Overall, these results are consistent with a role for HELZ2 in IFN-mediated suppression of DENV at a post-entry step. These findings are consistent with our results for HELZ2 in control of HCV, where we also found that HELZ2 activity occurred post-entry (Fusco et al., 2013).

#### Nuclear receptor interactions

**Figure 5:**
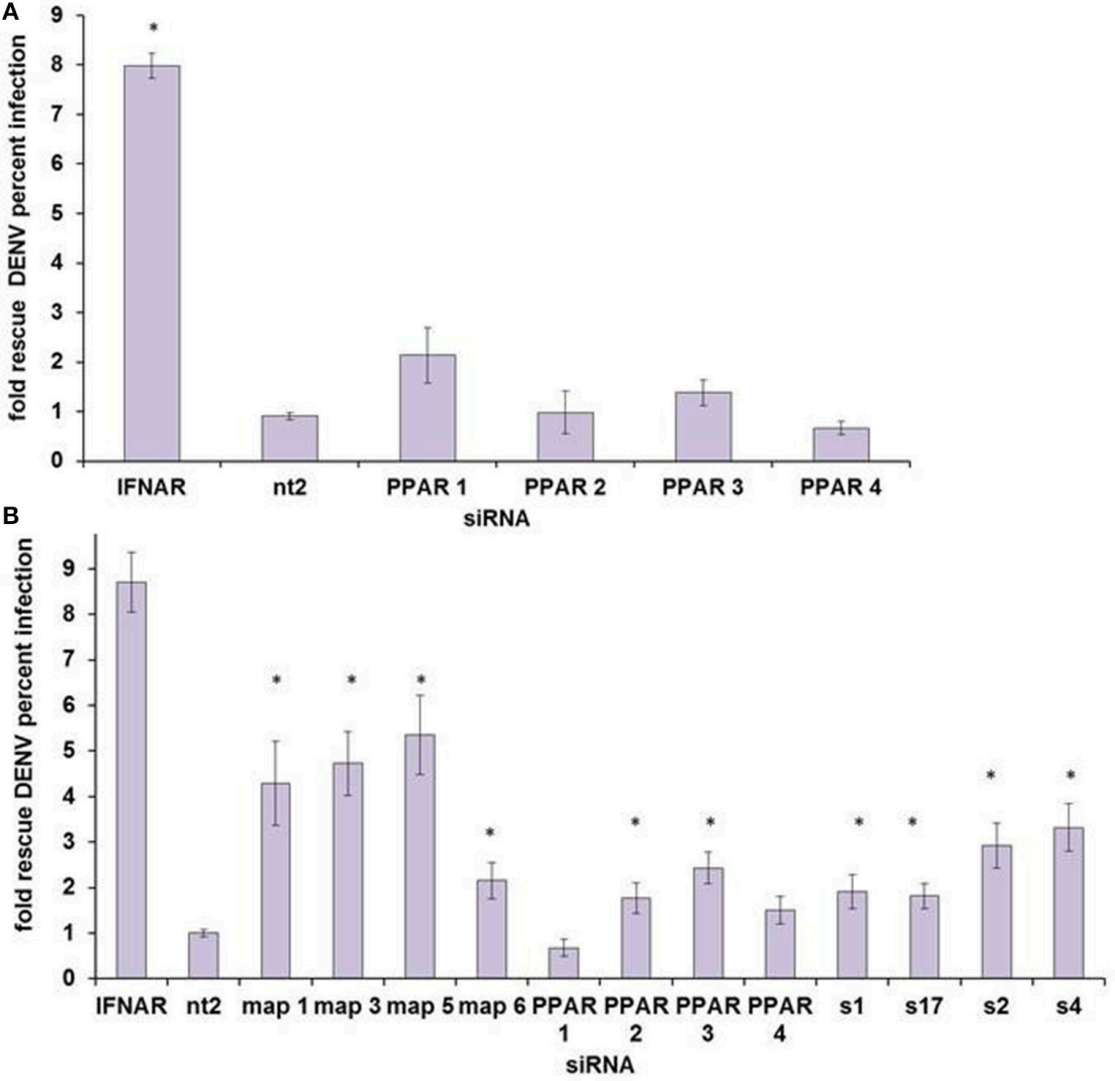
**PPARα does not appear to be the HELZ2-IFN Nuclear Receptor. (A)** HeLa cells were transfected with four unique siRNAs targeting PPARα, without any significant rescue of DENV from IFN as measured by percent infected cells, using IF as in Figure 1. **(B)** Huh7.5.1 cells were transfected with four unique siRNAs targeting PPARα, and two of four siRNAs led to significant rescue of DENV from IFN. Knockdown of two other nuclear-receptor family genes, MAP2K4 (map) and SLC27A2 (s) led to rescue of DENV from IFN for four of four unique siRNA duplexes. ^*^p < 0.05 compared to control.

We then sought to determine which, if any, nuclear receptor is the mediator of HELZ2-IFN antiviral effects. We initially examined PPARα, as there is some data supporting an antiviral role for PPARs (Arnold et al., 2007; Hanley et al., 2010; Wakui et al., 2010; Goldwasser et al., 2011; Hanley and Viglianti, 2011; Huang et al., 2011; Yoon et al., 2011; Sehgal et al., 2012). PPARα knockdown did rescue DENV from IFN in HeLa cells in our screen, but this result did not reproduce in HeLa cells outside of screening conditions (Datasheet S1, Figure 5A). However, PPARα knockdown did rescue DENV from IFN in Huh7.5.1. cells, as did knockdown of two other nuclear receptor family genes, MAP2K4 and SLC27A2 (Figure 5B). The PPARα ligand fenofibrate did suppress proportionate HCV infection, in hepatocytes, and proportionate DENV infection, in HeLa cells, but it did so only with significant cytotoxicity, arguing against a meaningful antiviral effect (Figure S6). IFN treatment did not upregulate PPARα mRNA or mRNA of the PPARα target gene, CPT1, though treatment with positive control fenofibrate did, albeit weakly, in the Huh7.5.1 cell line (Figure S7). Overall, these findings did not support a clear role for PPARα as the major HELZ2-IFN nuclear receptor. To better understand the mechanism of HELZ2 antiviral effects, we therefore turned our attention to identifying additional factors that interact with HELZ2 during IFN stimulation.

#### DNA interactions

**Figure 6:**
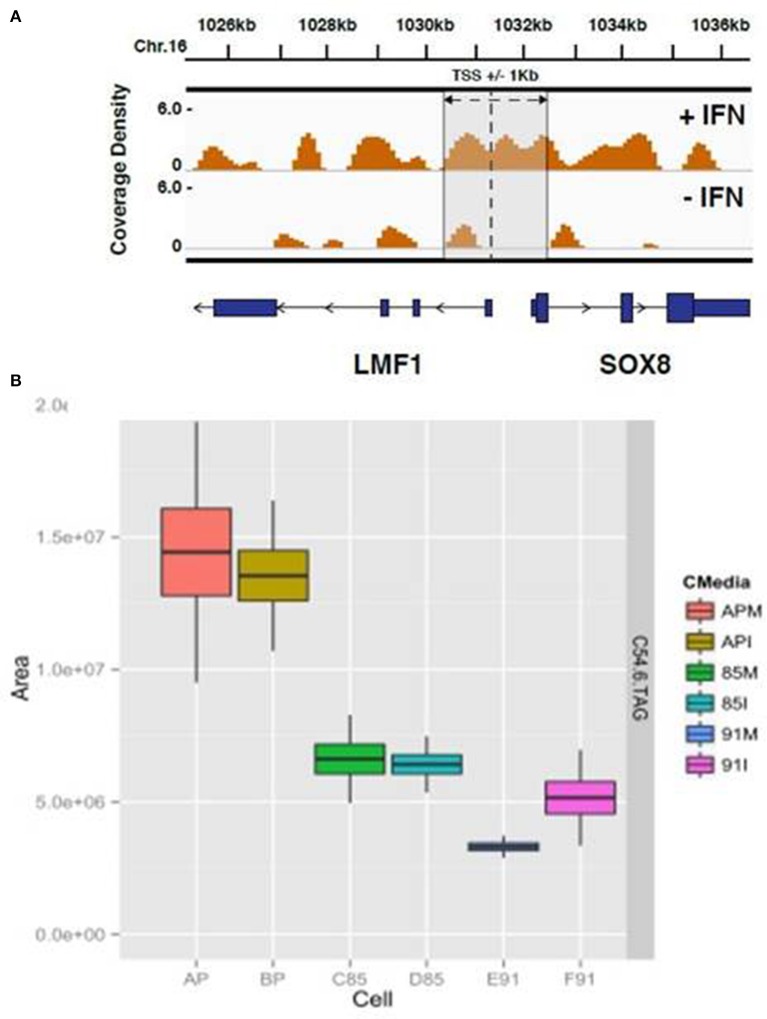
**HELZ2 CHiP seq and Metabolomics identify HELZ2 as a lipid regulator. (A)** CHiP seq to identify DNA bound by HELZ2 during IFN stimulation in hepatocytes. Chromatin immunoprecipitation was performed using anti-HELZ2 antibody on Huh7.5.1 human hepatocyte nuclear extracts following 24 h treatment with IFN. Sequencing of DNA pulled down by anti-HELZ2 revealed enrichment for LMF1 transcription start site region sequences in the presence, but not absence, of IFN. LMF1 encodes lipase maturation factor 1, a protein involved in the maturation and transport of lipoprotein lipase through the secretory pathway. The plot indicates the chromasome location of enriched sequences detected by chromatin IP, harvested from cells with (top) and without (bottom) IFN treatment, on the x axis, and coverage density, or quantity of DNA detected per nucleotide, on the y axis. The vertical line indicates the location of the TSS for LMF1, and the annotated Sox8 TSS is within 1 kb. **(B)** Metabolomics to determine the lipid profile of HELZ2 knockdown cells. Huh7.5.1 hepatocytes stably transduced with lentivirus containing vector control (AP), shRNA targeting HELZ2 long and short isoforms (85) or shRNA targeting HELZ2 long isoform only (91) were treated with medium (M) or IFN (I) followed by intracellular lipid extraction and analysis using mass spectrometry. HELZ2 knockdown cells were found to contain a highly significant decrease in the triglyceride TAG 54:6 (number of carbons in the lipid acyl chain: number of double bonds in the lipid acyl chain), compared to vector controls, both in the presence and absence of IFN treatment. In the box, the central line is the median, the lower margin of the box is the first quartile, the upper margin of the box is the third quartile. The upper whisker extends from the hinge to the highest value that is within 1.5^*^IQR of the hinge, and the lower whisker extends from the hinge to the lowest value that is within 1.5^*^IQR of the hinge.

The HELZ2 sequence contains a Superfamily I DNA and/or RNA helicase domain, raising the possibility that HELZ2 may regulate a specific subset of genes. However, direct HELZ2-DNA interactions have not been characterized. Because HELZ2 protein levels appear to increase in the nucleus upon IFN stimulation (Figure 4E), we hypothesized that HELZ2 may bind a unique DNA population in the presence of IFN. To test this hypothesis we performed chromatin immunoprecipitation sequencing (CHiP seq), using HELZ2 immunoprecipitation (IP) in human hepatocytes in the presence and absence of IFN. Because predicted sites of HELZ2-DNA interaction during IFN stimulation in hepatocytes were unknown, we used H3K27 immunoprecipitation of ActA2 enhancer sites as a positive control for successful chromatin immunoprecipitation. These experiments revealed that HELZ2 bound a unique population of DNA in the presence, but not absence, of IFN. DNA bound by HELZ2 uniquely in the presence of IFN included closest genebody [Transcription Start Site (TSS) to transcription termination site (TTS), including all introns]/enhancer site for LMF1 (Lipase Maturation Factor 1), which encodes a chaperone protein that activates vascular lipases which themselves regulate triglyceride maturation and processing (Figure 6A; Peterfy, 2012). Because we had identified HELZ2 interactions with lipid regulators, and HELZ2 is known to interact with nuclear receptors that regulate intracellular lipid state, we proceeded to examine the effect of HELZ2 expression on intracellular lipid state. It is important to note that DENV infection has been found to both require host lipids and to influence host lipid state, in clinical infection and in *in vitro* studies (Villar-Centeno et al., 2008; Heaton and Randall, 2010, 2011; Heaton et al., 2010; Gutsche et al., 2011; Duran et al., 2015; Martin-Acebes et al., 2016). Thus we hypothesized that IFN-mediated activation of HELZ2, and its downstream gene targets, could effectively flip a switch, converting host intracellular lipid state from “pro” to “anti” viral.

#### Role in intracellular lipid processing

We treated Huh7.5.1 cells with IFN or mock, then collected supernatant and intracellular lysate for mass spectrometry (MS) for high resolution lipid analysis (Rhee et al., 2011, 2013). Supernatant samples revealed limited variability among cell types, but intracellular lysate samples revealed major differences for several lipids when comparing HELZ2 knockdown vs. vector control cells. These differences included a highly significant decrease in the triglyceride TAG 54:6 (number of carbons in the lipid acyl chain: number of double bonds in the lipid acyl chain; Figure 6B). TAG54.6 a representative lipid, showing some of the most striking change comparing knockdown cells to vector controls, among many TAGs which were lower in HELZ2 knockdown cells vs. vector controls (Datasheet S4). Based on our findings that (1) HELZ2 knockdown cells have low triglyceride levels and (2) HELZ2 CHiP seq detected HELZ2:LMF1 interaction, we postulated that HELZ2 must interact with a lipid-regulating nuclear receptor. Since we had not found strong evidence supporting a role for PPARα as the HELZ2-IFN NR, we explored previously published datasets supporting the role of other NRs as potential regulators of the immune activation state and intracellular lipid status. In this search we found that the NR AHR fit these criteria. AHR was also the only other known ligand-activated transcription factor/nuclear receptor identified by bioinformatics analysis of our DENV IEG list (Figure 1E). We thus proceeded to explore a possible role of AHR as an interferon effector/HELZ2 interactor.

#### Re-examination of HELZ2—NR interactions: aryl hydrocarbon receptor

**Figure 7:**
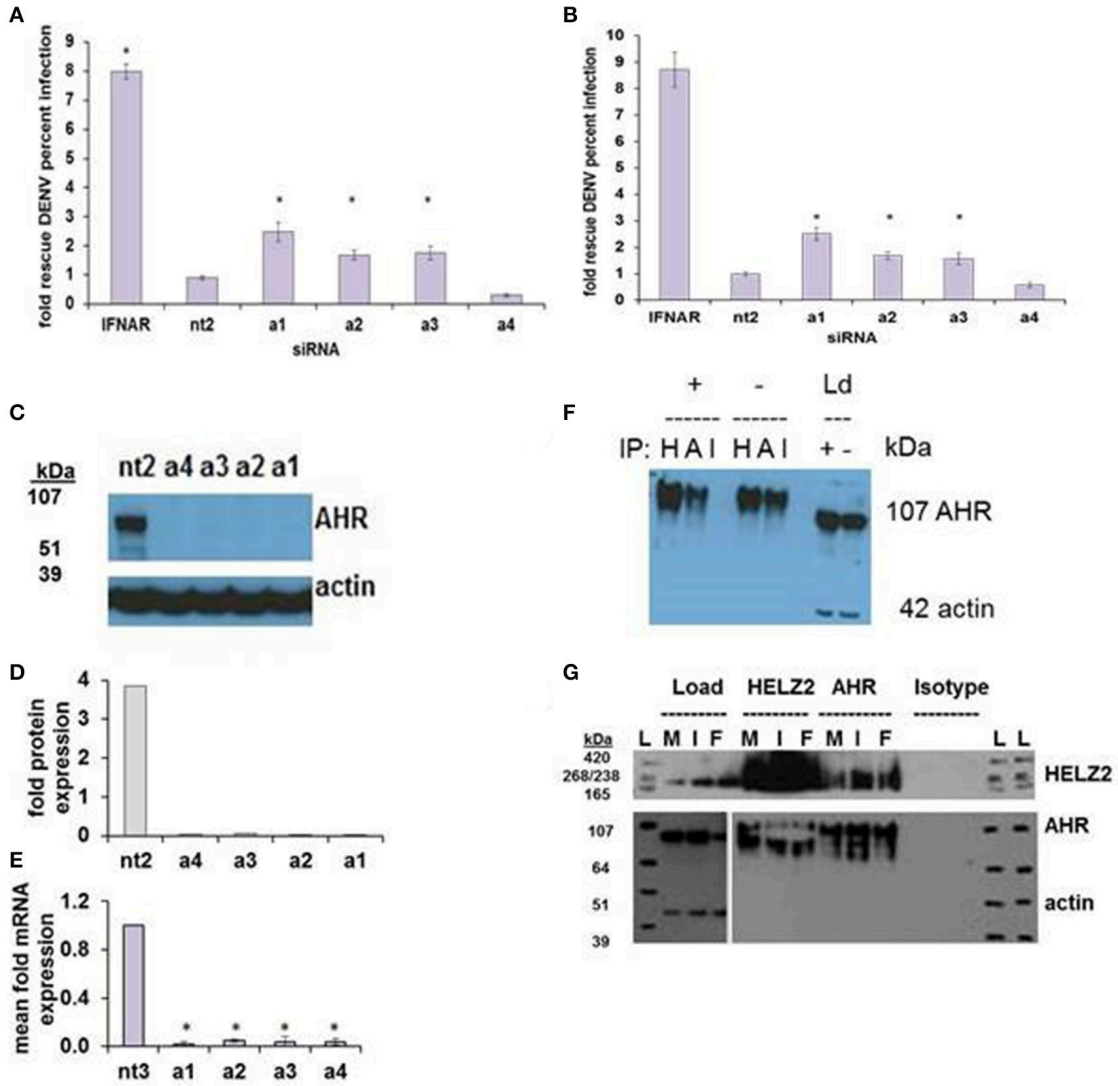
**AHR is an IFN Effector Gene and Interacts with HELZ2**. RNAi-mediated knockdown of AHR with four unique siRNAs leads to weak but consistent rescue of DENV from IFN in HeLa cells **(A)** and Huh7.5.1 hepatocytes **(B)**. **(C)** Knockdown of AHR by the four unique siRNAs from **(A)** was validated at the protein level using western blot **(C)** with quantification **(D)** and at the mRNA level using qRT PCR **(E)**. **(F)** Co-immunoprecipitation of AHR with HELZ2 (H) and AHR (A) antibodies and isotype control (I) was performed in Huh7.5.1 cells treated with IFN 1,600 IU/ml × 24 h or mock, followed by immunoblot for AHR. Load controls (Ld) were run in parallel, and immunoblotted for AHR and actin **(G)**. Co-immunoprecipitation was repeated in the setting of mock (M), IFN 1,600 IUml (I), or the AHR agonist FICZ 0.9 um (F) × 24 h, using HELZ2 and AHR antibodies for IP, followed by HELZ2 and AHR immunoblot. Load controls were stained for HELZ2, AHR, and actin. Ladder is indicated by L. ^*^p < 0.05 compared to control.

**Figure 8:**
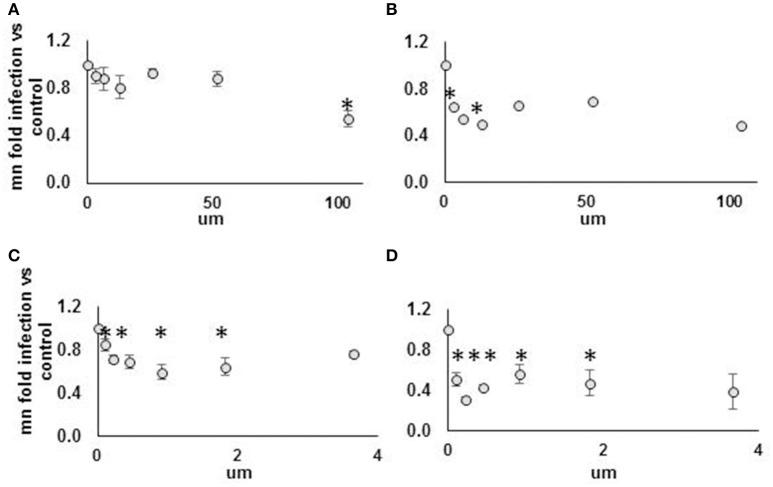
**Strong but not weak AHR agonist exhibits antiviral effect against DENV NGC2 in Huh7.5.1 cells**. Huh7.5.1 cells were plated then, 24 h later, treated with AHR agonist suspended in DMSO vs. DMSO alone, at indicated concentration, with or without submaximal dose IFN (25 IU/ml), for 24 h. Cells were then infected with DENV NGC2 at MOI of 1, followed by 48 h incubation, then fixation, permeabilization, and staining for DENV, followed by quantitative microscopy for cell counts and percent infected cells. Fold infection, vs. mock-treated cells, is shown. For the WEAK AGONIST (ITE), 0 um concentration, the % infected cells for three unique experiments were 18, 29, 40. For the STRONG AGONIST (FICZ), 0 um concentration, the % infected cells for three unique experiments were 20, 30, 32. Treatment groups are: **(A)** Weak agonist (ITE), no IFN. **(B)** Weak agonist (ITE), submax IFN. **(C)** Strong agonist (FICZ), no IFN. **(D)** Strong agonist (FICZ), submaximal IFN. ^*^p < 0.05 compared to control.

AHR knockdown in HeLa cells rescued DENV from IFN for three of four siRNA duplexes tested (Figure 7A). In hepatocytes, AHR knockdown rescued DENV from IFN for the same three siRNA duplexes that rescued DENV from IFN in HeLA cells, identifying AHR as a HeLa and hepatocyte DENV IEG (Figure 7B). AHR mRNA (Figures 7C,D) and protein knockdown (Figure 7E) were validated in HeLa cells. We then proceeded to look for evidence of HELZ2: AHR protein: protein interactions. Using HELZ2 Ab for immunoprecipitation (IP), it was possible to detect both HELZ2 and AHR, but not isotype control, by immunoblot (IB). Conversely, using AHR Ab for immunoprecipitation (IP), it was possible to detect both AHR and HELZ2, but not isotype control, by IB. The HELZ2:AHR interaction appeared to be conserved during treatment with both IFN and the AHR ligand FICZ (6-Formylindolo(3,2-b)carbazole) (Figures 7F,G). We next tested FICZ, which is a strong AHR ligand, and ITE (2-(1′H-indole-3′-carbonyl)-thiazole-4-carboxylic acid methyl ester), a weak AHR ligand, for antiviral effects against DENV, using the same pretreatment model used in our IFN experiments. Interestingly, FICZ, but not ITE, pretreatment of hepatocytes led to a 40% reduction of percent DENV infected cells (Figure 8) with minimal cytotoxicity (Figure S8). Furthermore, FICZ did potentiate the antiviral dose of submaximal IFN, though the dynamic range of these experiments was limited (Figure 8). To determine whether the antiviral effect of an AHR ligand was HELZ2 dependent, we then repeated the FICZ/ITE antiviral experiments in hepatocytes with stable knockdown of HELZ2-long and HELZ2 both isoforms and found that HELZ2 knockdown does trend toward abrogation of the anti-DENV effects of FICZ though this effect was not statistically significant (Figures S9, S10). Because FICZ activates AHR, which interacts with HELZ2, and both AHR and HELZ2 knockdown are associated with a decrease in TAGs, we hypothesized that FICZ antiviral effects may be due to a change in intracellular lipid composition. To test this hypothesis, we measured TAG levels in hepatocytes treated for 24 h with FICZ vs. DMSO using two separate methods (Datasheet S3, Supplemental Methods File). For the first method, we treated human hepatocytes with FICZ at the dose we found to have the greatest antiviral effect (Figure 8C, 920 um), vs. equal volume of DMSO, followed by a commercial assay for crude measurement of triacylglycerols (TAGs; BioVision K622). Using this assay, we did not identify any enhancement (or decrease) of TAGs during FICZ treatment, compared to DMSO (Figure S11A). At the same time, we treated human hepatocytes with FICZ (920 um), vs. equal volume of DMSO and harvested intracellular lysate for high content mass spectrometry, a much more sensitive assay for TAG levels, and also the technique used for the HELZ2 knockdown metabolite studies presented above (Figure 6B). Interestingly, these studies revealed that FICZ treatment does enhance the levels of a subset of TAGs which are also decreased by HELZ2 knockdown (Figure S11, Datasheet S4). This effect was most significant for TAG 58:9 and TAG 60:10. We have included the complete TAG datasets for both HELZ2 knockdown cells vs. vector controls as well as FICZ vs. DMSO (Datasheet S4).

**Figure 9:**
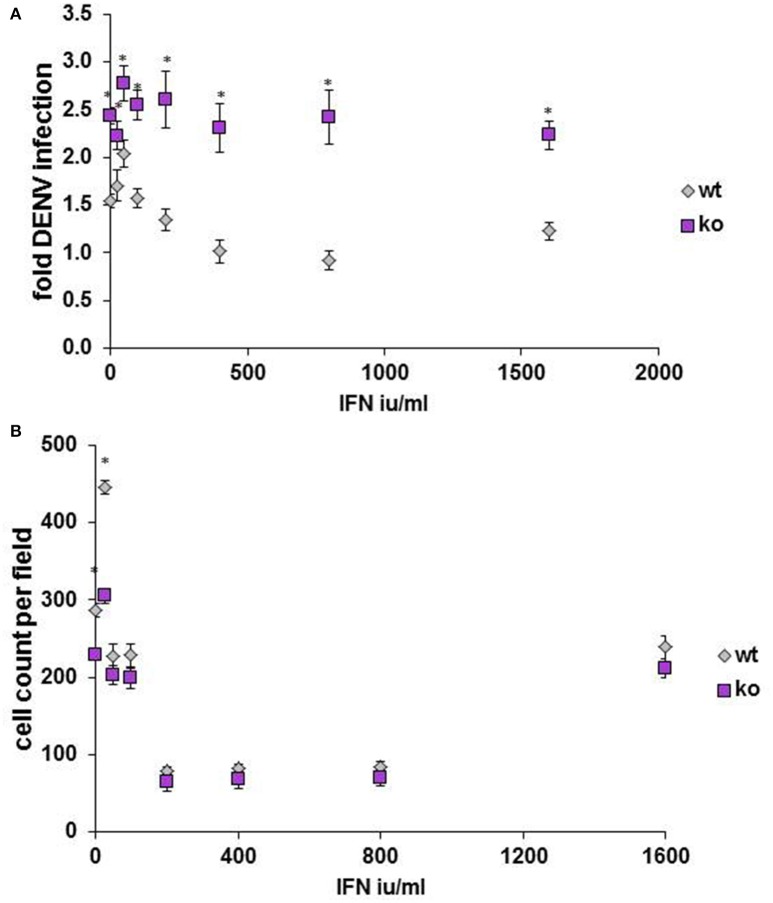
**Primary bone marrow derived macrophages of HELZ2 knockout mice display enhanced DENV infectivity compared to wild type controls**. Age and sex matched HELZ2 knockout and WT mice were euthanized followed by isolation of primary bone marrow derived macrophages. Cells were plated in 96 well black clear bottomed plates then, the next day, treated with IFN or mock, then, the next day, infected with DENV NGC2, followed by 48 h incubation. Cells were then fixed, permeabilized, stained, imaged, and analyzed for percent DENV infection. Graphs depict pooled data from three separate experiments which include a total of 7 WT mice and 6 K/O mice. **(A)** Indicates DENV fold infection. **(B)** Indicates cell count per field. ^*^p < 0.05 compared to control.

Finally, to determine whether our findings might be relevant in *in vivo* models, HELZ2 knockout mouse (Yoshino et al., 2014) primary bone marrow derived macrophages (BMDM) were tested for DENV infectivity, compared to primary BMDM from wild type (WT) controls. Consistent with a role for HELZ2 in IFN-mediated suppression of DENV, these cells exhibited both enhanced DENV infection and IFN resistance, compared to age matched wild type controls (Figure 9). Furthermore, wild type, but not knockout, mice exhibited enhanced sensitivity to murine, vs. human, IFN as measured by percent infection in the presence of IFN treatment (Figure S12).

## Discussion

Roughly half of the world population lives in regions at risk for DENV infection, and medical countermeasures for DENV are lacking. Furthermore, factors predicting severe DENV remain unclear. We have here attempted to help clarify which host factors participate in the IFN-mediated response to DENV. Our hope is that these factors, the DENV IEGs, may serve as (1) possible eventual targets for host directed antiviral design and/or (2) candidate biomarkers for study in variable DENV clinical outcomes.

In this study, we have performed a targeted RNAi screen seeking genes required for IFN to suppress both HCV and DENV, leading to identification of 56 HCV/DENV IEGs. These HCV/DENV IEGs included multiple nuclear receptor interactors (HELZ2, MAP2K4, SLC27A2, HSP90AA1, HSP90AA2), prompting further examination of IEG—nuclear receptor interactions. HELZ2 target gene knockdown was validated at the mRNA and protein levels, and IEG status confirmed outside of screening conditions. Impact of IFN on HELZ2 promoter and protein localization was then examined, revealing that IFN activates both HELZ2 isoforms, and IFN stimulation is associated with increased levels of nuclear HELZ2. Lifecycles assays measuring effect of HELZ2 knockdown on DENV via early-infection RNA ISH, qRT PCR, western blot, immunofluorescence, and supernatant plaque assay reveal that HELZ2 appears to exert anti-DENV activity from early infection onward. In our prior study of HELZ2 effect on HCV, we found that HELZ2 does not affect entry, as measured by a pseudoparticle assay, but does affect post-entry lifecycle stages (Fusco et al., 2013). While we have not performed DENV pseudoparticle studies for direct comparison, overall our findings for both DENV and HCV appear to be consistent in identifying a role for HELZ2 in suppression of multiple viral lifecycle stages, including production of viral RNA and protein production as well as viral release. Because HELZ2 is a nuclear receptor co-activator, we then sought to determine whether nuclear receptor agonists themselves suppressed DENV. We initially examined PPARα since potential antiviral and immunomodulatory activity of PPARα agonists during viral infection have been reported (Goldwasser et al., 2011; Sehgal et al., 2012), and PPARα agonists have been reported to reverse IFN refractoriness during flaviviral infection (Read et al., 2015). However, in our hands, agonists of the NR PPARα decreased virus replication but only in the setting of severe cytotoxicity.

We thus sought additional information to identify the NR with which HELZ2 interacts during IFN stimulation, using CHiP seq and high resolution lipid mass spectrometry. CHiP seq revealed HELZ2 interaction (either direct or indirect) with lipase maturation factor 1, whose gene product regulates TAG metabolism through lipoprotein lipases (Peterfy, 2012), during IFN stimulation. Metabolomic studies revealed that HELZ2 knockdown cells exhibit significant decrease of intracellular triglyceride subsets. Because HELZ2 appears to regulate triglycerides based on both CHiP seq and metabolomic data, we hypothesized that IFN-stimulated HELZ2 activates a NR that then alters triglyceride levels. Studies of mice with knockout of the nuclear receptor AHR, a known immunoregulatory nuclear receptor, have decreased triglycerides compared to wild type controls (Minami et al., 2008). Furthermore, CHiP seq experiments in MCF-7 human breast cancer cells treated with the exogenous AHR agonist TCDD (2,3,7,8-tetrachlorodibenzo-*p*-dioxin) identified AHR interactions with DNA from genes in the triglyceride pathway (Lo and Matthews, 2012). In their study of AHR DNA binding by CHiP seq, Lo et al. did not find that AHR binds HELZ2 DNA, in human breast cancer cells (without IFN stimulation; Lo and Matthews, 2012). However, they did find that AHR binds to SLC27A2, which contains a known AHRE (aryl hydrocarbon response element), and is another one of our top IEG hits for both HCV and DENV (Fusco et al., 2013). Furthermore, in both its liganded and non-liganded states, AHR binds to the chaperone protein HSP90 (Hankinson, 1995; Meyer and Perdew, 1999; Bell and Poland, 2000; Chang et al., 2014; Tsuji et al., 2014), which also scored as a top IEG hit in both our HCV and DENV screens. We therefore sought to determine whether, in hepatocytes, HELZ2 and AHR might interact to mediate IFN's antiviral effects, and found that (1) AHR knockdown led to weak but reproducible rescue of DENV from IFN, (2) immunoprecipitation (IP) with anti-HELZ2 detected AHR, and IP with anti-AHR detected HELZ2, and (3) a strong AHR agonist (FICZ, formylindolo[3,2-b]carbazole) suppressed DENV in the absence of cytotoxicity. FICZ is a tryptophan degradation product, induced both by light- dependent and -independent pathways (Smirnova et al., 2016), and an endogenous AHR ligand (Wincent et al., 2009). Interestingly, a role for type I IFNs in tryptophan degradation has been well described, but has not previously included FICZ. IFN beta induces indoleamine 2,3-dioxygenase (IDO), the enzyme responsible for tryptophan degradation into kynurenine and other metabolites (not FICZ), which have been implicated in IFN immune response, including that observed during IFN treatment of multiple sclerosis (Meyer et al., 1992; Durastanti et al., 2011). Furthermore, IDO activity induced by IFN gamma has been described to have antiviral properties against the DNA virus HSV-2 (Adams et al., 2004). Consistent with these findings of IFN-mediated tryptophan degradation, human sera from DENV infected patients have been found to have increased levels of IDO mRNA expression and IDO activity, compared to uninfected controls, eliciting the hypothesis that IDO and tryptophan catabolism contribute to the immune response to DENV infections *in vivo* (Becerra et al., 2009).

In contrast to IDO, the enzymes responsible for converting tryptophan into FICZ have not been reported to be IFN inducible. Likewise, there are no prior reports, to our knowledge, citing a direct role for IFN in *FICZ* production from tryptophan. However, there have been reports of FICZ production in skin affected by vitiligo (Schallreuter et al., 2012), a disease characterized by IFN alpha hyperproduction (Bertolotti et al., 2014). The mechanism through which FICZ might mediate antiviral effects is also not clear, though it is possible that FICZ binding to AHR prompts AHR translocation to the nucleus, where it then interacts with HELZ2 to alter lipid related gene activation. In support of this hypothesis, we found that FICZ treatment, at doses with highest anti-DENV effect, does augment a subset of TAGs which appear to be HELZ2 dependent. These findings raise the possibility that, in response to IFN, HELZ2 is upregulated and then the IEG HSP90 chaperones HELZ2 and activated AHR to the nucleus where AHR activates the fatty acid transporter/IEG SCL27A2 and the intracellular lipid/triglyceride milieu is altered, potentially from a “proviral” to an “antiviral” state. The AHR agonist FICZ may potentiate this antiviral process by increasing nuclear levels of AHR, as engagement of FICZ and other AHR ligands with AHR has been shown to prompt nuclear translocation (Figure 3A of Kostyuk et al., 2012; Tsuji et al., 2014). Future detailed studies of these HELZ2, SLC27A2, AHR, and lipid-virus interactions, beyond the scope of this paper, will be required to verify these hypotheses. Overall, our findings suggest that there is a conserved set of genes required for IFN-mediated suppression across at least two flaviviridae, enriched for nuclear receptor interactors such as the ISG HELZ2, which appear to modulate the intracellular triglyceride lipid milieu.

Limitations of our approach include the lack of an unbiased search for HELZ2-NR interactions, which may have identified additional NRs, and may have prompted discovery of more potent antiviral compounds. For this reason, unbiased studies of HELZ2 binding factors, in the presence and absence of IFN, merit attention in future studies. However, we postulate that the findings presented in this study do provide an initial step toward a platform of IFN-derived HDAV discovery. Refinement of this approach, including more extensive searches for compounds that activate HELZ2/HELZ pathways are likely to provide antiviral activity below the common viral subversion point of STAT1/2. Such compounds will likely be beneficial both for their antiviral activity and, perhaps equally importantly, for their ability to help normalize cytokine signal transduction during viral infections. Furthermore, the broad-acting potential of IFN-related compounds may provide badly needed placeholder solutions, pending distribution of more definitive countermeasures, during epidemics of acute viral infections. Finally, by defining key factors that dictate host IFN-mediated control of viral infections, we may be able to identify biomarkers to stratify clinical risk for viral pathology. Such biomarkers might delineate virus infection, or reactivation, risk posed by normal genetic variants as well as relative risk posed by the increasing number of immunomodulatory biologic drugs now progressing through clinical trials for treatment of diseases such as malignancy and autoimmune disorders.

## Methods

### Cell culture

Huh7.5.1 hepatoma cells (a gift from F. Chisari, Scripps Research Institute, La Jolla, CA.) and HeLa MAGI cells (a gift from A. Brass, U. Mass. Worcester, Worcester, MA.) were maintained at 37°C in humidified air containing 5% CO_2_ in Dulbecco's modified Eagle's medium (DMEM;Cellgro) supplemented with 15% (volume/volume) fetal bovine serum (FBS;Gibco, #10437) and 2 mM L-glutamine (Gibco). Medium was prepared on a weekly basis to maintain maximum L-glutamine activity. C636 mosquito salivary gland cells (ATCC CRL-1660) were cultured in 10% EMEM (ATCC #30-2003) supplemented with 10% (volume/volume) fetal bovine serum (FBS; Gibco #10437) and 2 mM L-glutamine (Gibco) with no antibiotics. Cells were maintained at 28°C with 5% CO_2_.

### Virus propagation

Dengue New Guinea C 2 was purchased from ATCC (ATCC VR1584). Virus was propagated by inoculating C636 mosquito cells with 1 ml viral stock for 1 h, followed by addition of 9 ml EMEM with 2% FBS with no antibiotics, at 28°C. Viral supernatant was harvested 4–8 days following inoculation, and clarified by centrifugation at 1,500 rpm × 15 min at 4°C. Intracellular virus was harvested by scraping remaining cells into 1 ml remaining medium, and then alternately exposing cell mixture to dry ice and 37°C water bath for periods of 5 min, repeated 3–5 times, followed by centrifugation at 1,500 rpm × 15 min and aliquoted for storage at −80°C.

### DENV IEG screen

DENV IEG Screen was performed at the Harvard ICCB-L Screening Facility. Four unique siRNA duplexes (siGenome, Dharmacon) targeting each of 120 HCV IEGs (Fusco et al., 2013) were arrayed in plate format. After quality control testing (Z prime), screening was performed in 384 well plate format. HeLa cells were reverse transfected with siRNA using oligofectamine (Life Technologies 12252-011). Forty-eight hours after transfection, transfection medium was removed by aspiration and cells were treated with IFN 1,600 IU/ml. Twenty-four hours following IFN treatment, IFN medium was removed by aspiration and cells were infected with DENV NGC2 at an MOI of 1. Cells were then incubated for 48 h, followed by fixation in 4% paraformaldehyde (Sigma), permeabilization in 0.2% Triton (Fisher BP151-500), then staining in primary anti-dengue envelope antibody at 4°C overnight (ATCC HB-114). The following day, cells were rinsed, followed by staining in secondary goat-anti-mouse antibody (Invitrogen A11001 Alexa Fluor 488 goat anti mouse antibody) for 1 h at room temperature. Cells were then rinsed followed by staining with Hoechst anti-DNA stain (Sigma H6024). Plates were then imaged at 10X magnification on a Molecular Devices ImageXpress Micro high-content screening microscope system, and analyzed using MetaXpress technology for number of DENV-infected cells, total number of cells, and calculation of percent infected cells.

### Out-of-screen confirmation

Select DENV IEGs were confirmed out-of-screen. siGENOME siRNA sets-of-four (upgrades) were purchased from Dharmacon/GE Lifesciences and reverse transfections performed, as described above, in HeLa and Huh7.5.1 hepatocytes.

### Validation of siRNA target gene knockdown

For HELZ2 and AHR, knockdown of target gene product was confirmed at the mRNA and protein level using qRT PCR and western blot, respectively. For qRT PCR, HeLa cells were reverse transfected in 24 well dishes, followed by RNA isolation using QIAshreddor (Cat. 79654), then RNeasy RNA isolation kit including genomic DNA eliminator columns (Cat. 74134). RNA was then reverse transcribed to DNA (Applied Biosystems High Capacity cDNA reverse transcription kit, #4368813), followed by qRT PCR using human TaqMAN probes against HELZ2 (huHELZ2 Hs 00375699) and AHR (huAHR Hs 00169233), normalized using GAPDH huGAP (Hs 02758991). For western blot, HeLa cells were reverse transfected in 24 well dishes followed by protein isolation using Cell Signaling Protein Isolation buffer with PMSF, then protein quantification using either Bradford or micro BCA method (Thermo Scientific #23235), then western blot. Western blot quantification was performed using image analysis software developed in our laboratory, GELQuant, which is available to the public upon request.

### Total RNA isolation and quantitative RT PCR (qRT-PCR)

Intracellular total cellular and viral RNA was isolated using QIAshredder™ (Qiagen), followed by removal of genomic DNA and RNA isolation using RNEasy plus kits (Qiagen), all according to manufacterer's protocols. RNA was stored at minus 80°C, then reverse transcribed by random priming using the High capacity cDNA reverse transcription kit (Applied Biosciences) and quantified by real-time PCR using the DyNAmo HS SYBR green qPCR kit (Finnzyme;Espoo, Finland). qRT-PCR results were interpreted using delta/delta CT (PMID 11328886), using GAPDH as an internal control. Gene specific primers are provided in Datasheet S5.

### Western blots

#### Drug treatment

Cells were plated 24–48 h prior to drug treatment. When the cells were confluent, they were given fresh medium containing either 1,600 IU/mL interferon, 10 um fenofibrate (Sigma F6020), or no drug as a negative control. The cells were incubated in their respective medium at 37°C for 24 h.

#### Protein isolation

The cells were harvested using trypsin and pelleted at 500 g for 5 min. The pellets were then resuspended in ice-cold PBS and pelleted again at 500 g for 5 min. Subcellular fractionation was then performed using the Thermo Scientific Subcellular Protein Fractionation Kit [product #78840] per the manufacturer's instructions. Protein samples were stored at minus 80°C.

#### Western blot

Protein concentration was determined by Bradford assay kit (BioRad ##5000116). Twenty microliters of the least concentrated sample was then used for western blot analysis; the more concentrated samples were normalized to this first sample such that the same amount of protein was loaded for each (20 ug). Deionized water was added as needed to bring each sample volume to 20 uL. Samples were mixed with 7 uL NuPage sample reducing agent and blue dye mixture and incubated for 10 min at 80°C to denature the proteins. Samples were then run on a NuPage gel in 1x MES SDS running buffer at 180 V for 1 h, with LifeTechnologies' HiMark™ Pre-Stained High Molecular Weight Protein Standard.

A tank transfer onto nitrocellulose membranes was performed using 1x NuPage Transfer buffer with 20% methanol at 4°C and 30 V for 2 h. Membranes were then blocked for 1 h at room temperature in 5% milk in 1x TBST. The membranes were then cut horizontally and primary antibodies were added, in 5% milk in 1x TBST.

**Table:** 

**Primary Antibodies**
– Anti HELZ2, 1:1,000 (rabbit): abcam ab129781
– Anti Na+/K+ ATPase, 1:2,000 (rabbit) rabbit polyclonal, abcam ab58475
Anti SP1, 1:2,000 (mouse) ms, abcam ab77441
– Anti β-actin, 1:10,000 (mouse) mc, ms, sigma A2228-2004 L
– Anti-tubulin, 1:2,000 (mouse) ms anti beta II tubulin abcam Ab 28035
– Anti-histone H3, 1:80,000 (rabbit) rbt anti histone abcam ab1791

Membranes were incubated with primary antibodies overnight at 4°C with gentle mixing. They were then washed 5 times for 5 min each wash at room temperature with gentle mixing in 1x TBST. Following the washes, the membranes were blocked for 1 h at room temperature in their respective secondary antibodies (see diagram above) at a concentration of 1:5,000 in 5% milk in 1x TBST. Five more 5 min washes in 1x TBST were performed prior to development.

ThermoScientific SuperSignal chemiluminescent substrate was required to visualize HELZ2, SP1, and Na+/K+ ATPase. For actin, tubulin, and histone H3, standard ThermoScientific ECL substrate was used. Membranes were allowed to sit in their respective substrate mixtures for 5 min before development. Proteins were visualized on blue X-Ray films; for each membrane, exposures of 2, 5, 10, 30 s and 2 min were performed. When bands appeared were too dark for quantitative analysis even after 2 s, repeat exposures were performed after allowing the membranes to sit in the dark room for several minutes to allow luminescence to fade.

### Quantitative analysis

#### Obtaining images

Bands were scanned using an Epson scanner on the color photo setting. Quantitative analysis was performed on the resulting images using an original program. Each image was 3,504 × 2,550 pixels with horizontal and vertical resolution each 300 dpi.

#### Determining background signal

Background color density was determined manually for each scan by selecting a rectangular portion of the image that was known not to have been exposed to any signal. Color density was calculated by dividing the total color value of the region by the area of the region.

#### Validating bands

Rectangular regions containing bands were manually selected from each scan for analysis. Each selected region was divided by the program into rectangular subregions of equal width containing the bands themselves. A vertical cross-section from the center of each subregion was taken for manual analysis, and the program plotted the color values for each pixel of the cross section moving from top to bottom. In order to be considered valid for quantitative analysis, a cross-section needed to display a clear maximum color-value at some vertical coordinate; cross-sections that rose to a color-value plateau instead were considered to have saturated the X-Ray film's capacity to accurately record a signal and were quantified from a film developed with a shorter exposure.

#### Quantifying bands

For all bands considered quantifiable, the program moved from left to right generating vertical cross-sections at every horizontal coordinate. The program then determined the peak color-value for each vertical cross section. These peak values were then averaged to produce the final “signal” value for the band. These signal values were assumed to correspond to the amount of protein generating a given band and were used in analysis.

#### Normalization of bands

HELZ2 signal values were normalized to their respective β–actin signal values. For each compartment, the HELZ2:actin ratio for the cells that received no drug treatment was considered to be baseline, and the HELZ2:actin ratios for the interferon- and fenofibrate-treated cells were normalized to the non-treated value to produce fold-change values. Normalization was not performed between compartments as β–actin is not necessarily expected to be present in the same concentration in different subcellular compartments. When three replicates of the experiment had been performed, these fold-change values were averaged and standard deviations of the mean were calculated for the interferon and fenofibrate conditions. Fold-change values were not calculated for the cytoskeleton as HELZ2 signal was not significantly above background for these samples.

### Bioinformatics

Screen hits were analyzed using Ingenuity Pathway Analysis (IPA). HELZ2 promoter search for IFN stimulated response elements (ISREs) was performed using the Position Specific Scoring Matrix (PSSM) AGTTTCAGTTTC (Tsukahara et al., 2006), and HOMER motif discovery software.

ISRE(IRF)/ThioMac-LPS-Expression(GSE23622)/Homer, BestGuess:ISRE(IRF)/ThioMac-LPS-xpression(GSE23622)/Homer(1.000)10.512507 −1.194927e+02 0

87179.0,1722.0,789.4,104.0,0.00e+00

0.653653653653654 0.001001001001001 0.229229229229229

0.116116116116116

   0.008 0.288 0.676 0.028

   0.001 0.003 0.001 0.995

   0.001 0.027 0.001 0.971

   0.001 0.001 0.025 0.973

0.003003003003003 0.975975975975976 0.019019019019019

0.002002002002002

   0.583 0.007 0.24 0.17

0.0790790790790791 0.393393393393393 0.471471471471471

0.056056056056056

   0.016 0.001 0.001 0.982

   0.001 0.001 0.004 0.994

0.00899100899100899 0.044955044955045 0.003996003996004

0.942057942057942

   0.001 0.881 0.01 0.108

FIMO discovered 3 ISREs in the Helz-Beta promoter at marginal significance.

A *p*-value cutoff of 0.0001 was employed, and revealed 3 ISREs in Helz2-beta promoter. No ISREs were discovered for Helz2-alpha at *p*-value cutoff of 0.0001. The actual corrected *p*-values (*q*-value, aka FDR) ranged from 0.143 to 0.581 (Datasheet S5).

These putative ISREs are highlighted in yellow in Datasheets S2 and S5.

V$ISRE_01|2058 (+)|cAGTTTCGATTCtgg (*q* = 0.581).

V$ISRE_01|5166 (−)|tggAAAGTGAAATTg (*q* = 0.143).

### HELZ2 isoform specific promoter reporter experiments

The unique promoter for each HELZ2 isoform was cloned into a luciferase expression reporter. HELZ2 isoform-specific promoter luciferase constructs 5′-flanking regions of the human HELZ2 gene [−13,347/+25 base pairs (bp) of long isoform, −19,832/+23 bp of short isoform] were inserted into KpnI/NheI sites of the pGL4.10 luciferase reporter vector (Promega) employing a recombinant engineering strategy as previously described (Liu et al., 2003; Tomaru et al., 2009). Sequence information was obtained from the UCSC genome browser. The reporter for each isoform was transfected into separate groups of Huh7.5.1 hepatocytes or HeLa cells, followed by treatment with IFN or drug, then luciferase development at indicated time point.

### Co-immunoprecipitation protocol

Huh7.5.1 cells were plated in 10 cm dishes, incubated overnight, then treated with mock, IFN 1,600 IU/ml, or 0.9 um FICZ.

#### Antibody binding to beads

Protein G beads (Novex by Life Technologies, #10003D, 30 mg/ml) and Protein A beads (Novex by Life Technologies, #10001D, 30 mg/ml) were then prepared in quantity adequate for 3.5 ul multiplied by (the number of samples multiplied by number of Ab per sample + 0.5). For example, for two Ab, two samples, 2 × 2 = 4 + 0.5 = 4.5 × 3.5 ul = 15.75 ul protein G beads. Protein G and A beads were combined into a single microfuge tube, followed by addition of 1 ml of 0.5% BSA, then placement onto magnet. Bead mixture was then divided into separate microfuge tubes for each antibody (500 ul/tube), followed by addition of 500 ul more BSA to each tube. Ten micrograms of appropriate antibody was then added to each tube and samples placed at 4°C shaker for 5–6 h. Antibodies used were HELZ2 (abcam 129781), AHR (enzo BML-SA550-0100), and isotype control (purified Armenian hamster IgG, eBiosciences # 14-4888-85).

#### Protein isolation and quantification

Each 10 cm dish was then rinsed in 1xPBS, trypsinized, then cells centrifuged at 2,000 rpm for 5 min, followed by aspiration of trypsin and pellet resuspension in cell signaling lysis buffer (190 ul dH20, 10 ul LB, 1 ul PMSF). Each sample was then incubated on ice for 30 min with vortexing every 10 min. Samples were then spun at 16,000 rcf at 4°C for 10 min, followed by collection of supernatant containing protein for further processing. Protein samples, maintained at all times on ice, were then quantified by the uBCA method.

#### Protein: Ab/bead incubation

Beads were then retrieved from 4°C rotator, placed on magnet and washed twice with 1 ml 0.5% BSA. Four hundred microliters CHiP incubation buffer was then added to each tube, and 200 ul of each Ab:bead mixture distributed to a new tube for incubation with the appropriate protein sample. One hundred micrograms of the appropriate protein was then added to each Ab:bead mixture, followed by filling to 1,500 ul total volume per tube, with CHiP incubation buffer. Samples were then incubated overnight at 4°C on shaker.

#### IP complex retrieval

The following morning, samples were retrieved from 4°C, then washed thrice for 5 min at 4°C in 1x PBS with 0.5% triton x-100. Beads were then replaced onto magnet, followed by aspiration of wash, then addition of 100 ul Laemmli buffer (Laemmli-BOLT LDS-#B0007 Novex), then heating to 100°C for 8 min then loading of 20 ul of each IP lysate for western blot. During wash steps, original protein samples were retrieved from −80 and 20 ug prepared for gel loading (20 ug protein, filled to 13 ul with H20, plus 2 ul 10X buffer, and 5 ul 4X SRA buffer), and heated to 100°C for 8 min. Western blot was then processed as above.

#### CHiP incubation buffer

1% triton x-100, 2 mM EDTA, 150 mM NaCL, 20 mM tris-HCL, pH 8.1.

### AHR agonist treatment experiments

ITE was obtained from Tocris (# 1803) and diluted to 10 um in DMSO. FICZ was obtained from ENZO (# BML-GR206-0100) and diluted to 0.3517 um in DMSO. Both reagents were stored at −20°C. Huh7.5.1 or HeLa cells were plated at 4,000 cells per well in 96 well black clear bottomed plates (Corning Costar #3603), followed 24 h later by treatment with AHR agonist with or without IFN for 24 h, then addition of virus concentrate (25 ul) on top of medium, then 48 h incubation, then fixation, permeabilization, and staining for imaging/analysis for percent infection.

### Metabolite extraction

Huh7.5.1 cells were plated in 10 cm dishes overnight then were treated with mock or IFN 1,600 IU/l for 24 h, followed by harvest of supernatant and cells for LC MS according to published methods for Intracellular Metabolite Extraction/Lipid Method (LPCs, LPEs, SMs, PIs, PEs, PCs, DAGs, TAGs) for 10 cm Dish (Rhee et al., 2011, 2013). Huh7.5.1 cells were plated at ~7,500,000 Cells in a 10 cm plate. When ready for extraction, media was siphoned, with aliquot of 1 ml stored in respectively labeled tubes, stored at −80°C, followed by extraction protocol. Ten microliters of conditioned media samples were deproteinized with 190 μl Lipid IS-IS [isopropanol (Sigma-Aldrich) containing 10 μg/mL of internal standards 17:1 Lyso-PC, 12:0, 13:0 PC, 17:0, 20:4 PC, and 20 μg/mL of 19:0 CE (Avanti)]. After vortexing, the samples were centrifuged at 20,000 g at 4°C for 15 min and supernatants were transferred to HPLC quality glass vials with inserts (MicroSolv).

Plates were *gently* immersed in optima grade HPLC H_2_O (Fisher Chemical) for 1–2 s. H_2_O was then siphoned off. Plates were then inverted (i.e., bottom-up), and Liquid Nitrogen poured directly onto under-surface of plate using Liquid Nitrogen-resistant basin. Liquid Nitrogen was then allowed to evaporate (5–10 s). Plate was then turned and 5 ml of ice-cold 2-Propanol (Fisher Chemical A461-212, Optima LC/MS) poured to evenly cover surface of 10 cm plate, which was left for approximately 15 s. Samples were then aspirated 3 × 1.25 ml into 3, respectively, labeled sample tubes (i.e., 3.75 ml total).

1.25 ml was then aspirated into collective (i.e., from each plate) 15 ml tube (pooled lysate), and after mixing (i.e., inverting and vortexing), 1.25 ml aspirated each into tubes labeled as “PL (pooled lysate) 1, PL2” etc. (PLs were intermittently spaced throughout the run to serve as a quality control). Samples were then spun at 14,000 rpm × 10 min at 4°C, then aliquotted in aliquots of 1 ml into conical Eppendorf tubes, labeled as above, transferred on dry ice, spun in a speedvac (Thermo Scientific) to dry down, and resuspended in 100 ul Lipid IS-IS. Finally, 65 ul was then placed into glass tubes for mass spectrometry.

### Metabolomic profiling

Deproteinized extracts were subjected to reverse phase chromatography using a HPLC C4 column (Grace) and mobile phases [mobile phase A: 10 mM Ammonium Acetate: Methanol: Acetic Acid (Sigma-Aldrich) (95:5:0.1, v:v:v); mobile phase B: 0.1% Acetic acid in Methanol (Sigma-Aldrich)]. The samples were injected directly onto the C4 column that was eluted at a flow rate of 350 μL/min with initial conditions of 80% mobile phase A and 20% mobile phase B for 2 min, followed by a 1 min linear gradient to 20% mobile phase A, then a 12 min linear gradient to 0% mobile phase A, maintained for 10 min, then a 1 min linear gradient to 80% mobile phase A, maintained for 9 min. Injection volume was 10 ul. The multiplexed liquid chromatography (LC) system comprised of a 1,200 Series pump (Agilent Technologies) and an HTS PAL autosampler (Leap Technologies) equipped with two injection ports and a column selection valve.

The LC system was connected to a 4,000 QTrap triple quadropole mass spectrometer (Applied Biosystems/Sciex) run in positive ion mode, in full scan mode (Q1 MS). MS analyses were carried out using electrospray ionization (ESI).

Metabolite peaks were integrated using Sciex MultiQuant software. All metabolite peaks were manually reviewed for peak quality in a blinded manner. In addition, pooled cellular extract samples were interspersed within each analytical run at standardized intervals every 10 injections, enabling the monitoring and correction for temporal drift in mass spectrometry performance. The nearest neighbor flanking pair of pooled plasma was used to normalize samples in a metabolite-by-metabolite manner. Internal standard peak areas were monitored for quality control and individual samples with peak areas differing from the group mean by more than two standard deviations were reanalyzed.

### CHiP seq

Huh7.5.1 cells were plated in 10 cm dishes at 80–90% confluence then treated with IFN or mock for 24 h, followed by nuclear extraction for CHiP seq.

#### Covaris sonication and ChIP protocol

*Preblock and binding of antibody to magnetic beads*. Dynal beads were washed in block solution then resuspended in block solution with antibody followed by overnight incubation at 4°C. Beads were then washed in block solution then resuspended in 0.5% BSA/sample. *Nuclei Preparation*. Cross-linked cells were thawed on ice, followed by isolation of nuclei using Covaris kit according to manufacturer's protocol, then shearing (Duty Cycle: 5%; Peak Incident Power: 140 Watts; Cycles/Burst: 200, Temp: 4°C). *Post-Shearing*. Sheared samples were transferred into cold 1.5 mL Lo-bind microcentrifuge tubes and centrifuged followed by transfer of supernatant containing sheared chromatin to a new cold 1.5 mL Lo-bind tube, removal of ~50 μL for Whole Cell Extract and storage at minus 20°C, and removal of ~900 μL for IP or storage at minus 80°C. *ChIP*. Antibody/Bead Solution was then added to each IP sample, followed by overnight incubation at 4°C. *Crosslink Reversal:* Beads were then precipitated with dynamag, then washed with Low salt buffer + protease inhibitor then High Salt buffer + protease inhibitor then 1X LiCl + protease inhibitor then 1X TE Buffer + 50 mM NaCl (100 uL/10 ml) followed by elution at 65°C for 45 min, with vortexing every 5 min, then centrifugation (to spin down beads) then removal of supernatant and transfer to new lo-bind microcentrifuge tube, and reversal of crosslinks at 65°C for 6 h. *Digestion of Cellular Protein and RNA was then performed using* RNase A followed by Proteinase K. DNA was then extracted with 400 μL Phenol:Chloroform:IsoamylOH. DNA concentrations were measured with Qubit.

### CHiP-seq sequencing and analysis

Sequencing was performed on Illumina HiSeq 2,500 instrument, resulting in ~40 million single-end 50 bp reads per sample. Reads were aligned against the hg19 reference genome using BWA (Li and Durbin, 2009). Alignments were filtered for uniquely mapped reads and alignment duplicates were removed. Input-normalized coverage tracks were generated using SPP (Kharchenko et al., 2008).

To identify potential protein binding sites, we determined regions of ChIP-Seq tag enrichment. In brief, we analyzed tag counts in a 1 Kb window over the chromosome length with the step of 200 bp and estimated statistical significance of enrichment of ChIP vs. input using negative binomial distribution, with the estimate of the mean based on the tag counts in input, and the size parameter (s) selected based on manual inspection of resulting peak calls. Regions of significant enrichment were generated by merging adjacent significantly enriched windows separated by 1 Kb or less. In addition, we called broad regions of enrichment using the peak caller SPP (Li and Durbin, 2009).

Since many of the peaks that were identified overlapped with transcriptional start sites (TSSs), we also compared input-normalized coverages over all annotated TSS ±1 Kb windows. This resulted in the identification of additional regions of enrichment not previously discovered through peak calling alone.

### Statistics

IEG hits were selected based upon 1.5x rescue of DENV infection, compared to non-targeting control, on 2 or more plates, for 2 or more siRNA duplexes. Out-of-screen confirmation siRNA transfection data was compared using means with standard error of the means (SEM). qRT PCR data was analyzed using the δδCT method, and data was compared using means with SEM. Students *t-*test (2 tailed, unequal variance) was used for determination of statistical significance.

## Ethics statement

This study was carried out in accordance with the recommendations of American Association for Laboratory Animal Care. The protocol was approved by the MGH IACUC Committee (2015N000185, DNF).

## Author contributions

DF, HP, DC, MB, JO, TT, SY, SK, SC, XS, and CY designed and performed the experiments. DF, HP, WL, DC, MB, KJ, AA, RS, JO, RG, TT, SY, TS, SK, SC, CY, XS, and RC analyzed and interpreted the data. DF wrote the manuscript then HP, WL, DC, MB, KJ, AA, RS, JO, RG, TT, SY, TS, SK, SC, XS, CY, and RC performed critical review and edits of the manuscript.

### Conflict of interest statement

The authors declare that the research was conducted in the absence of any commercial or financial relationships that could be construed as a potential conflict of interest.
