# Supplementary material for: HELZ2 Is an IFN Effector Mediating Suppression of Dengue Virus
Source: Front Microbiol. 2017 Feb 20;8:240. doi: 10.3389/fmicb.2017.00240 (PMC5316548; doi:10.3389/fmicb.2017.00240)
Supplement: Supplementary file 1 [file Presentation1.pptx]

## Slide 1
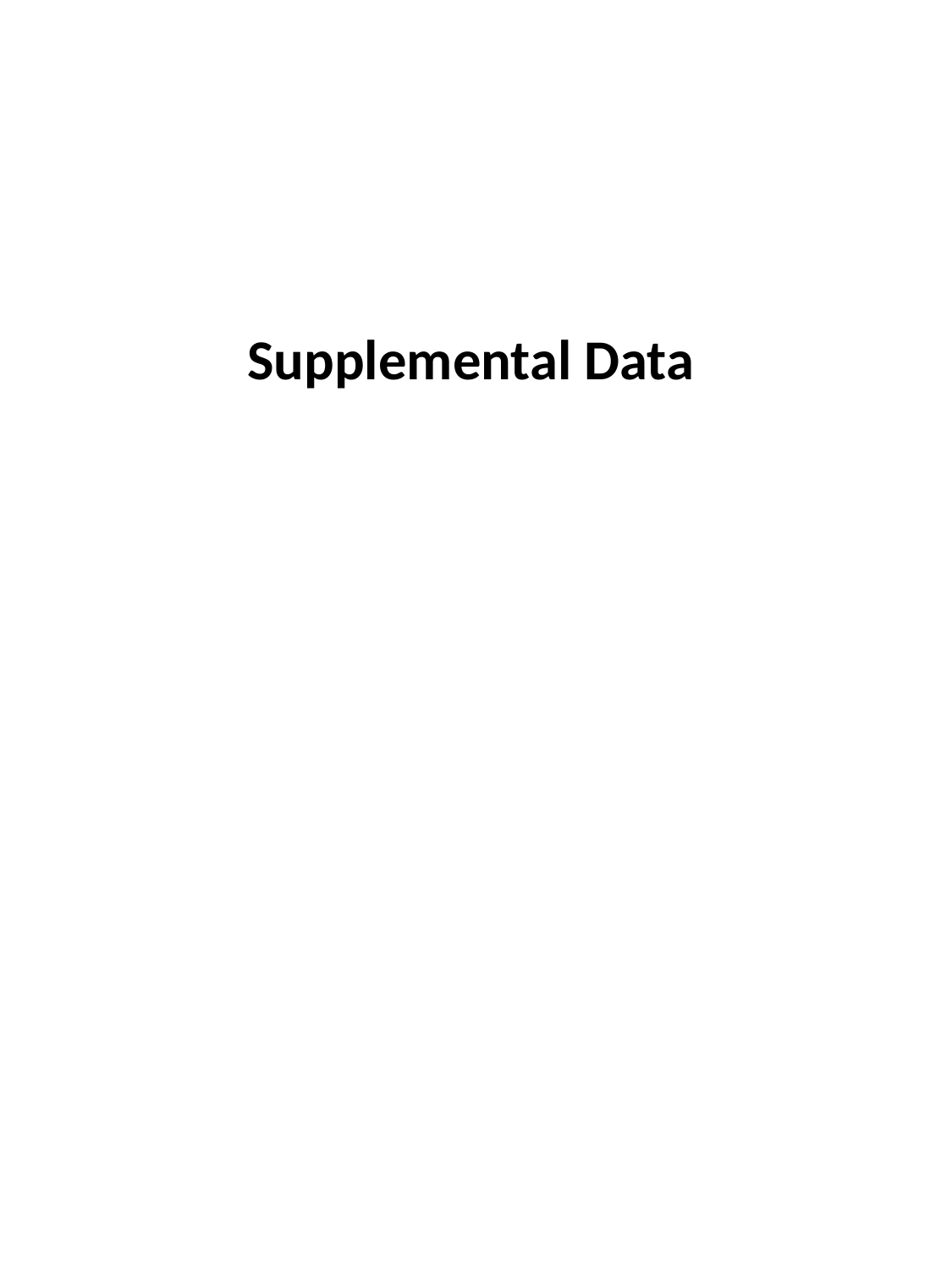

Supplemental Data

## Slide 2
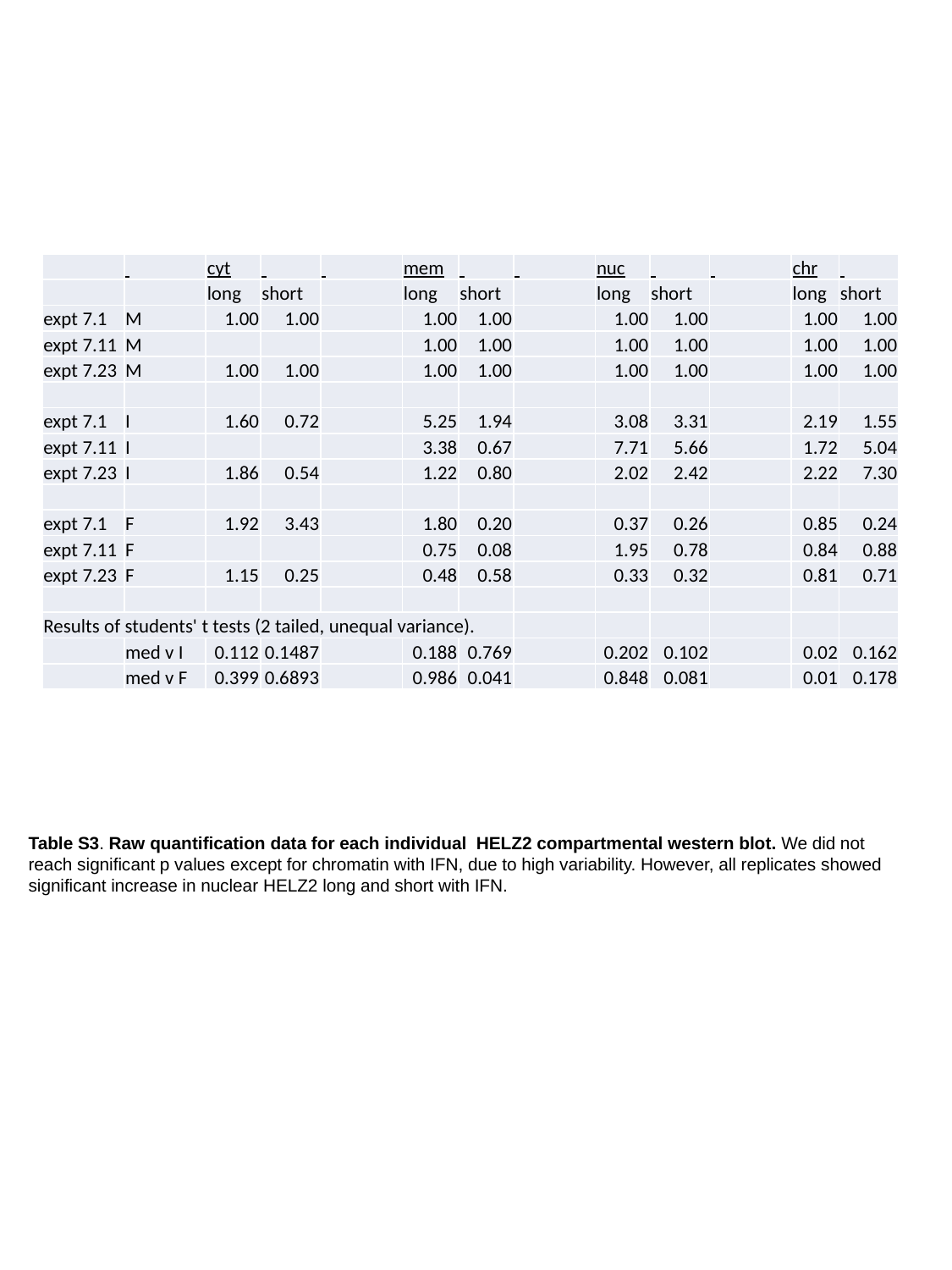

| | | cyt | | | mem | | | nuc | | | chr | |
| --- | --- | --- | --- | --- | --- | --- | --- | --- | --- | --- | --- | --- |
| | | long | short | | long | short | | long | short | | long | short |
| expt 7.1 | M | 1.00 | 1.00 | | 1.00 | 1.00 | | 1.00 | 1.00 | | 1.00 | 1.00 |
| expt 7.11 | M | | | | 1.00 | 1.00 | | 1.00 | 1.00 | | 1.00 | 1.00 |
| expt 7.23 | M | 1.00 | 1.00 | | 1.00 | 1.00 | | 1.00 | 1.00 | | 1.00 | 1.00 |
| | | | | | | | | | | | | |
| expt 7.1 | I | 1.60 | 0.72 | | 5.25 | 1.94 | | 3.08 | 3.31 | | 2.19 | 1.55 |
| expt 7.11 | I | | | | 3.38 | 0.67 | | 7.71 | 5.66 | | 1.72 | 5.04 |
| expt 7.23 | I | 1.86 | 0.54 | | 1.22 | 0.80 | | 2.02 | 2.42 | | 2.22 | 7.30 |
| | | | | | | | | | | | | |
| expt 7.1 | F | 1.92 | 3.43 | | 1.80 | 0.20 | | 0.37 | 0.26 | | 0.85 | 0.24 |
| expt 7.11 | F | | | | 0.75 | 0.08 | | 1.95 | 0.78 | | 0.84 | 0.88 |
| expt 7.23 | F | 1.15 | 0.25 | | 0.48 | 0.58 | | 0.33 | 0.32 | | 0.81 | 0.71 |
| | | | | | | | | | | | | |
| Results of students' t tests (2 tailed, unequal variance). | | | | | | | | | | | | |
| | med v I | 0.112 | 0.1487 | | 0.188 | 0.769 | | 0.202 | 0.102 | | 0.02 | 0.162 |
| | med v F | 0.399 | 0.6893 | | 0.986 | 0.041 | | 0.848 | 0.081 | | 0.01 | 0.178 |
Table S3. Raw quantification data for each individual HELZ2 compartmental western blot. We did not reach significant p values except for chromatin with IFN, due to high variability. However, all replicates showed significant increase in nuclear HELZ2 long and short with IFN.

## Slide 3
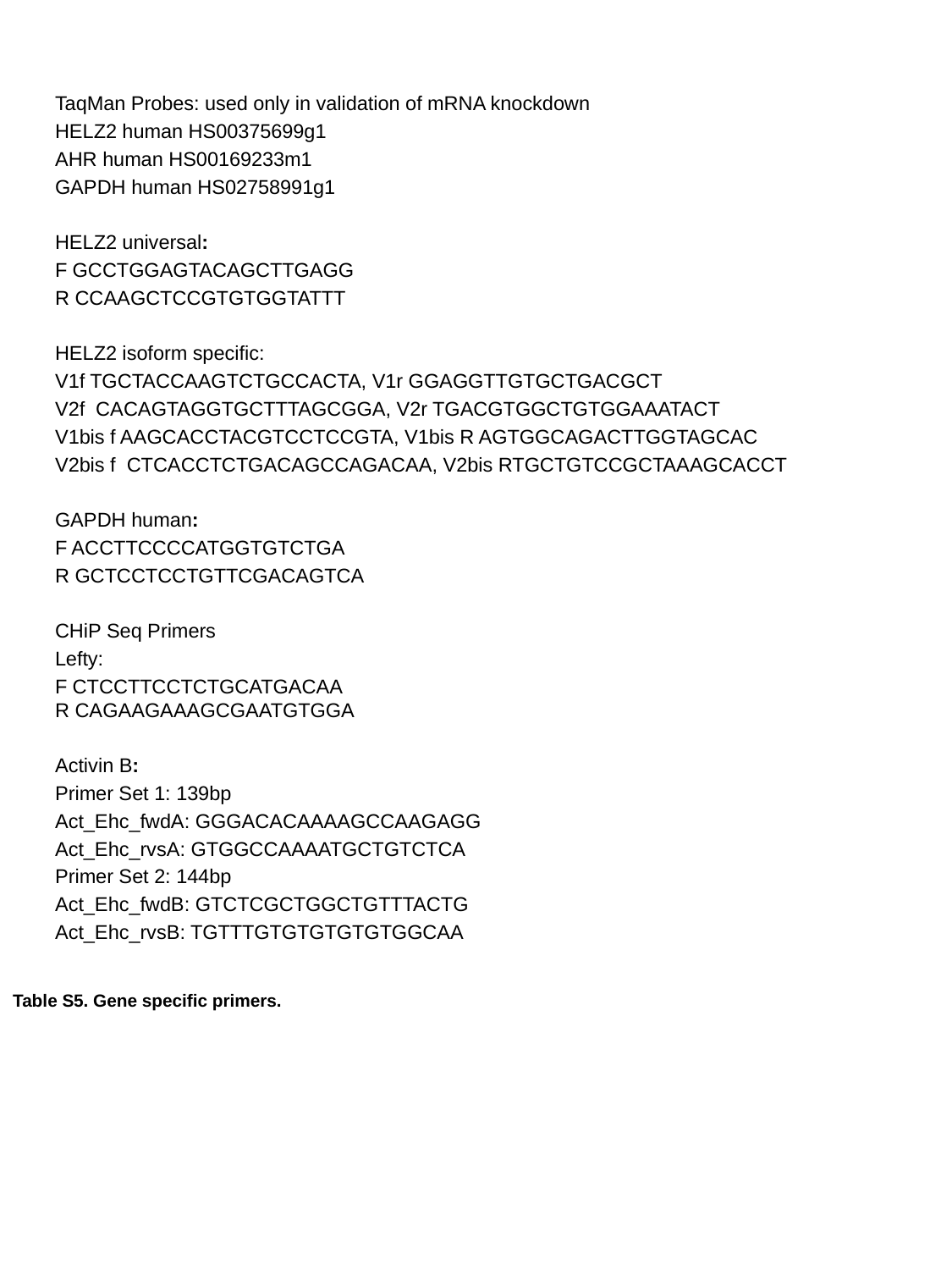

TaqMan Probes: used only in validation of mRNA knockdown
HELZ2 human HS00375699g1
AHR human HS00169233m1
GAPDH human HS02758991g1
HELZ2 universal:
F GCCTGGAGTACAGCTTGAGG
R CCAAGCTCCGTGTGGTATTT
HELZ2 isoform specific:
V1f TGCTACCAAGTCTGCCACTA, V1r GGAGGTTGTGCTGACGCT
V2f CACAGTAGGTGCTTTAGCGGA, V2r TGACGTGGCTGTGGAAATACT
V1bis f AAGCACCTACGTCCTCCGTA, V1bis R AGTGGCAGACTTGGTAGCAC
V2bis f CTCACCTCTGACAGCCAGACAA, V2bis RTGCTGTCCGCTAAAGCACCT
GAPDH human:
F ACCTTCCCCATGGTGTCTGA
R GCTCCTCCTGTTCGACAGTCA
CHiP Seq Primers
Lefty:
F CTCCTTCCTCTGCATGACAA
R CAGAAGAAAGCGAATGTGGA
Activin B:
Primer Set 1: 139bp
Act_Ehc_fwdA: GGGACACAAAAGCCAAGAGG
Act_Ehc_rvsA: GTGGCCAAAATGCTGTCTCA
Primer Set 2: 144bp
Act_Ehc_fwdB: GTCTCGCTGGCTGTTTACTG
Act_Ehc_rvsB: TGTTTGTGTGTGTGTGGCAA
# Table S5. Gene specific primers.

## Slide 4
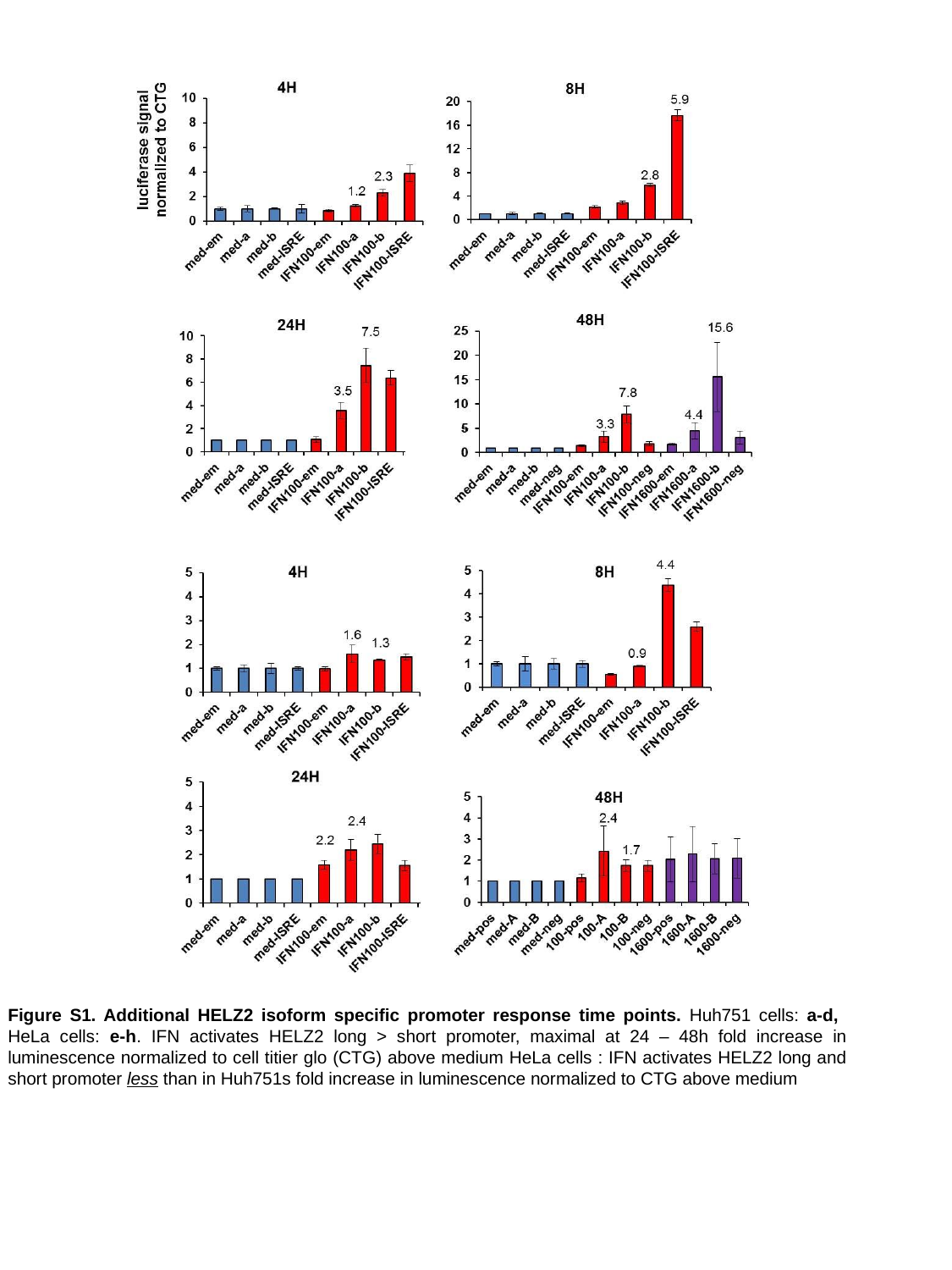

Figure S1. Additional HELZ2 isoform specific promoter response time points. Huh751 cells: a-d, HeLa cells: e-h. IFN activates HELZ2 long > short promoter, maximal at 24 – 48h fold increase in luminescence normalized to cell titier glo (CTG) above medium HeLa cells : IFN activates HELZ2 long and short promoter less than in Huh751s fold increase in luminescence normalized to CTG above medium

## Slide 5
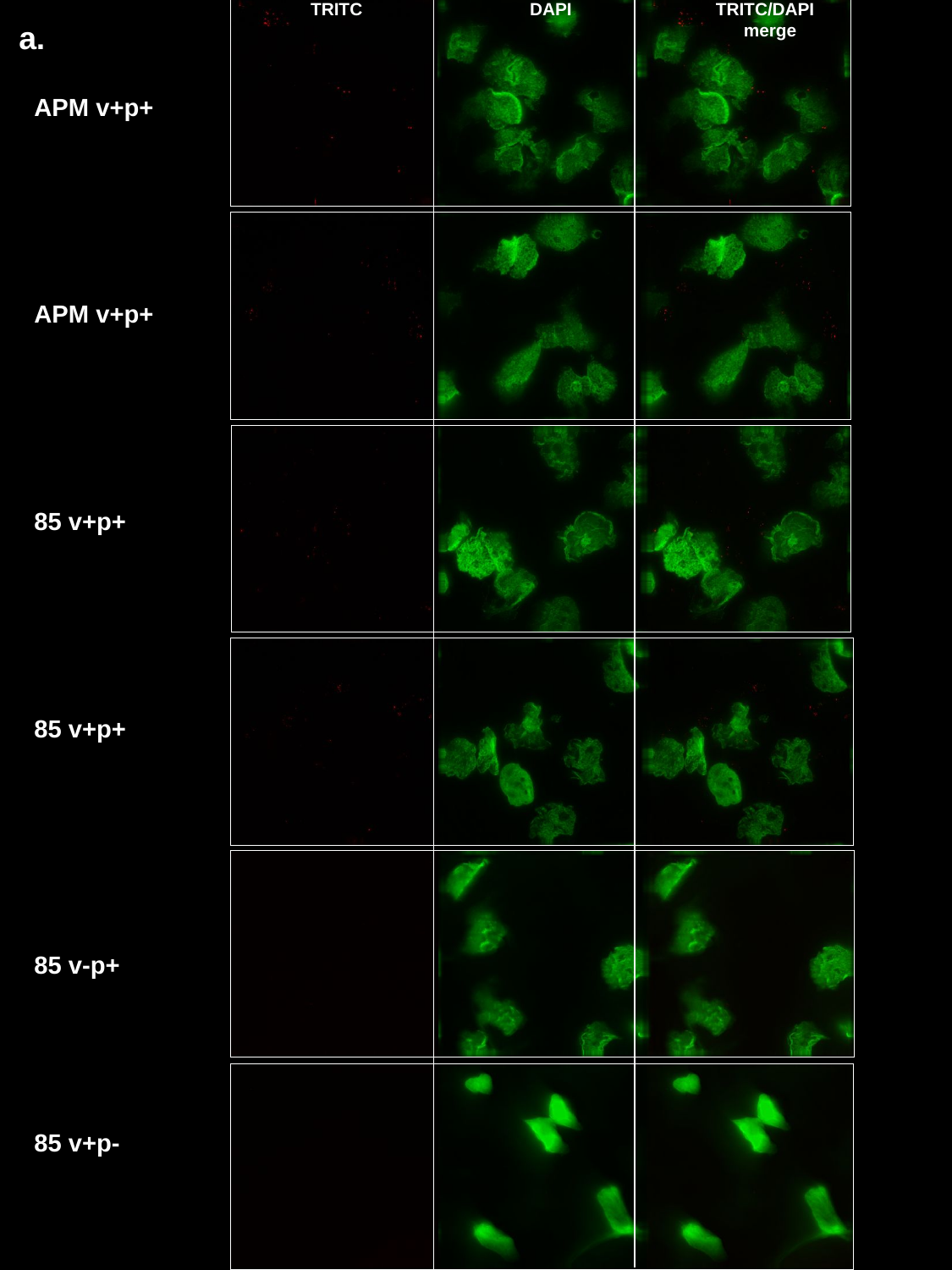

TRITC	 DAPI	 TRITC/DAPI
 merge
a.
APM v+p+
APM v+p+
85 v+p+
85 v+p+
85 v-p+
85 v+p-

## Slide 6
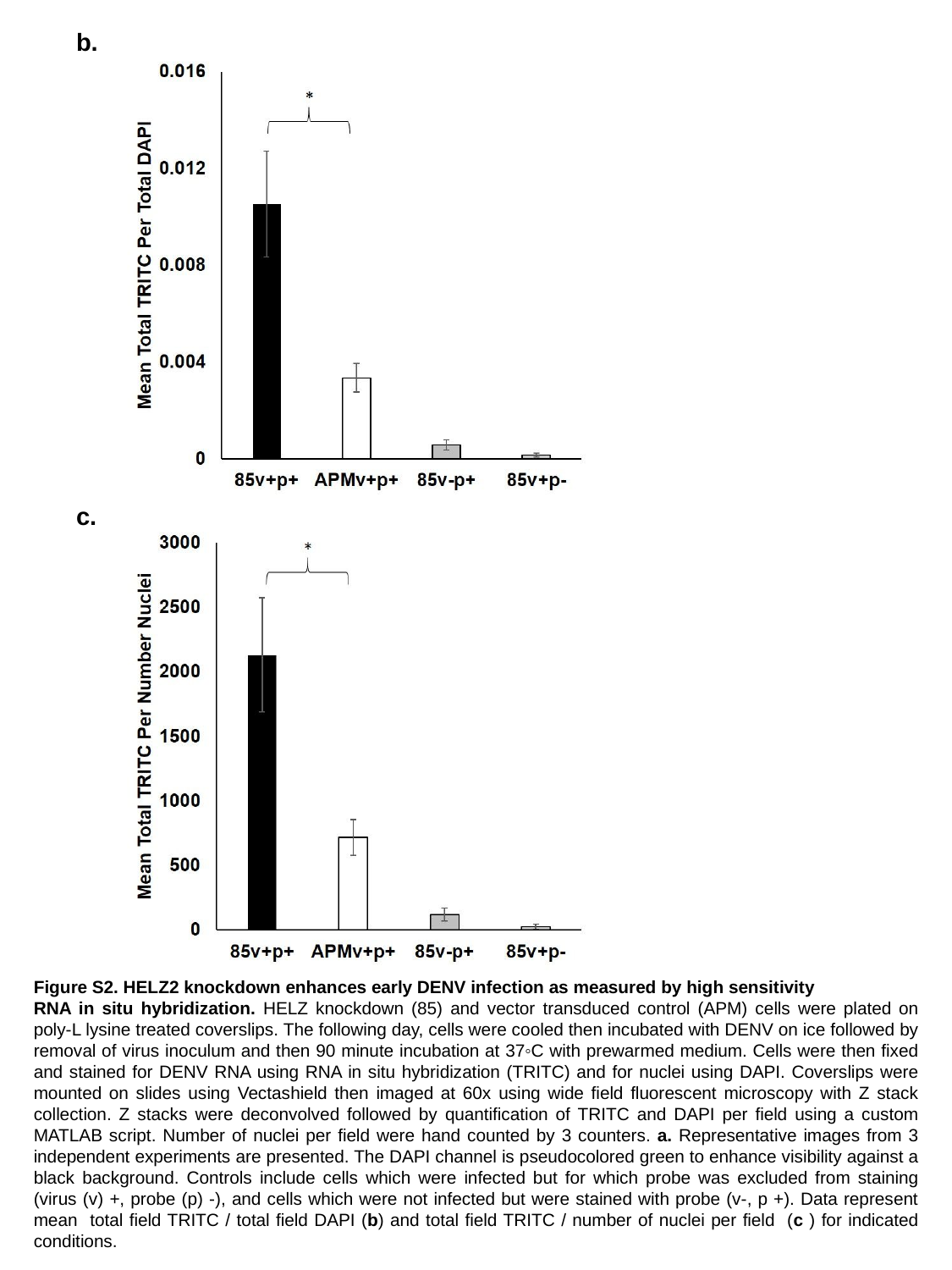

b.
c.
Figure S2. HELZ2 knockdown enhances early DENV infection as measured by high sensitivity
RNA in situ hybridization. HELZ knockdown (85) and vector transduced control (APM) cells were plated on poly-L lysine treated coverslips. The following day, cells were cooled then incubated with DENV on ice followed by removal of virus inoculum and then 90 minute incubation at 37◦C with prewarmed medium. Cells were then fixed and stained for DENV RNA using RNA in situ hybridization (TRITC) and for nuclei using DAPI. Coverslips were mounted on slides using Vectashield then imaged at 60x using wide field fluorescent microscopy with Z stack collection. Z stacks were deconvolved followed by quantification of TRITC and DAPI per field using a custom MATLAB script. Number of nuclei per field were hand counted by 3 counters. a. Representative images from 3 independent experiments are presented. The DAPI channel is pseudocolored green to enhance visibility against a black background. Controls include cells which were infected but for which probe was excluded from staining (virus (v) +, probe (p) -), and cells which were not infected but were stained with probe (v-, p +). Data represent mean total field TRITC / total field DAPI (b) and total field TRITC / number of nuclei per field (c ) for indicated conditions.

## Slide 7
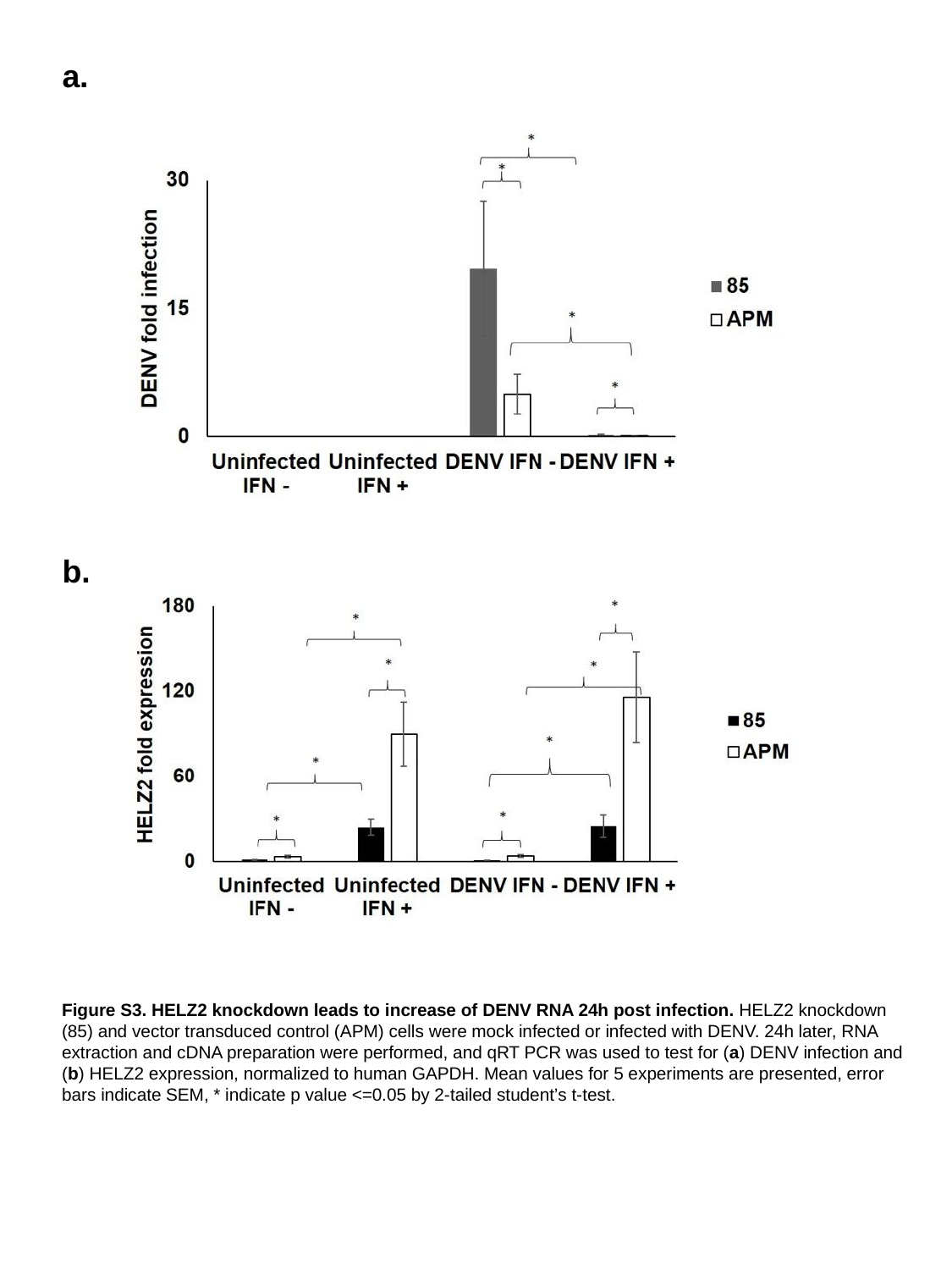

a.
b.
Figure S3. HELZ2 knockdown leads to increase of DENV RNA 24h post infection. HELZ2 knockdown (85) and vector transduced control (APM) cells were mock infected or infected with DENV. 24h later, RNA extraction and cDNA preparation were performed, and qRT PCR was used to test for (a) DENV infection and (b) HELZ2 expression, normalized to human GAPDH. Mean values for 5 experiments are presented, error bars indicate SEM, * indicate p value <=0.05 by 2-tailed student’s t-test.

## Slide 8
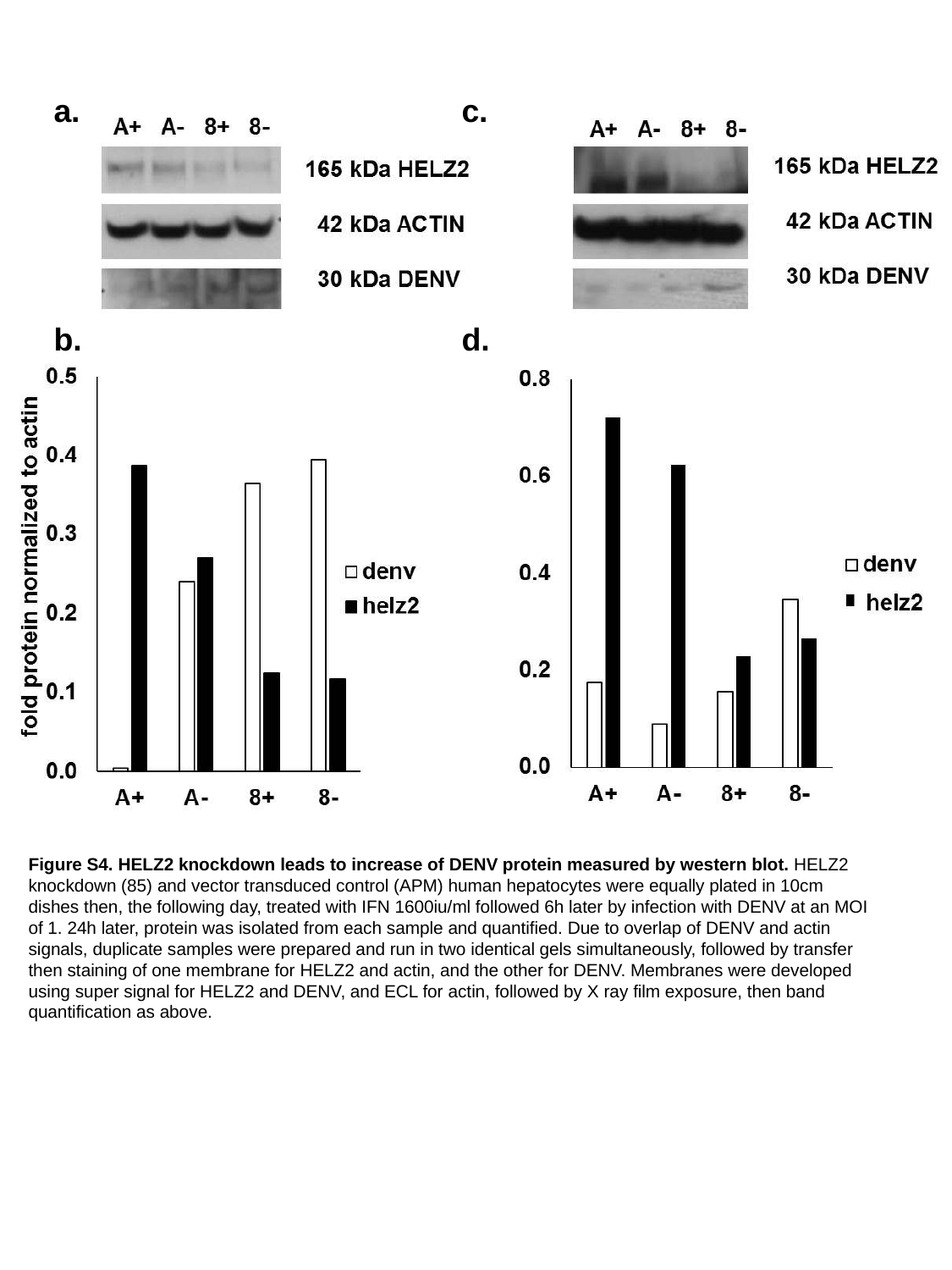

a.
b.
c.
d.
Figure S4. HELZ2 knockdown leads to increase of DENV protein measured by western blot. HELZ2 knockdown (85) and vector transduced control (APM) human hepatocytes were equally plated in 10cm dishes then, the following day, treated with IFN 1600iu/ml followed 6h later by infection with DENV at an MOI of 1. 24h later, protein was isolated from each sample and quantified. Due to overlap of DENV and actin signals, duplicate samples were prepared and run in two identical gels simultaneously, followed by transfer then staining of one membrane for HELZ2 and actin, and the other for DENV. Membranes were developed using super signal for HELZ2 and DENV, and ECL for actin, followed by X ray film exposure, then band quantification as above.

## Slide 9
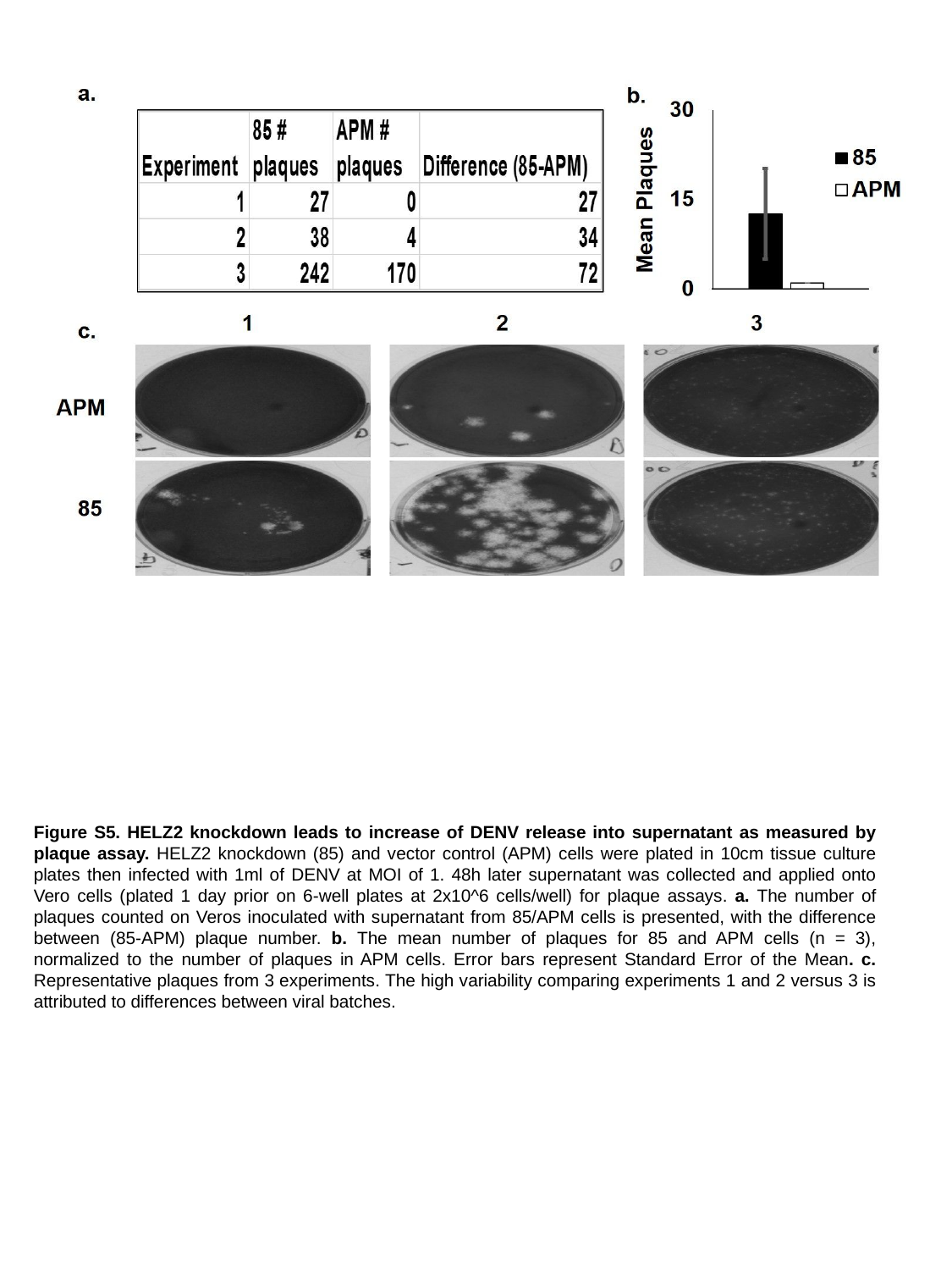

Figure S5. HELZ2 knockdown leads to increase of DENV release into supernatant as measured by plaque assay. HELZ2 knockdown (85) and vector control (APM) cells were plated in 10cm tissue culture plates then infected with 1ml of DENV at MOI of 1. 48h later supernatant was collected and applied onto Vero cells (plated 1 day prior on 6-well plates at 2x10^6 cells/well) for plaque assays. a. The number of plaques counted on Veros inoculated with supernatant from 85/APM cells is presented, with the difference between (85-APM) plaque number. b. The mean number of plaques for 85 and APM cells (n = 3), normalized to the number of plaques in APM cells. Error bars represent Standard Error of the Mean. c. Representative plaques from 3 experiments. The high variability comparing experiments 1 and 2 versus 3 is attributed to differences between viral batches.

## Slide 10
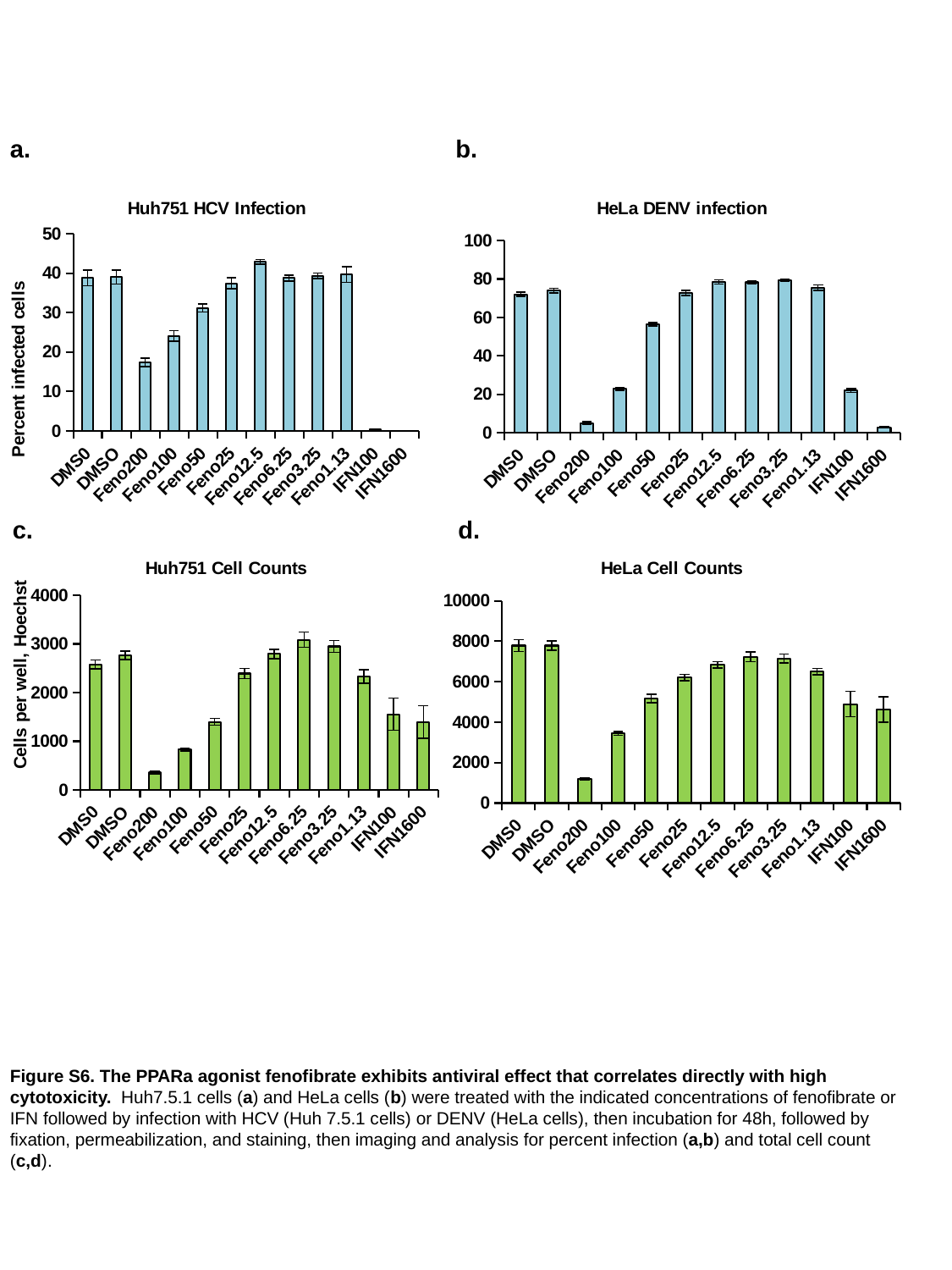

a. b.
### Chart: Huh751 HCV Infection
| Category | |
|---|---|
| DMS0 | 38.7773625 |
| DMSO | 39.004675 |
| Feno200 | 17.391612499999997 |
| Feno100 | 24.105375 |
| Feno50 | 31.1517875 |
| Feno25 | 37.437124999999995 |
| Feno12.5 | 42.873850000000004 |
| Feno6.25 | 38.76425 |
| Feno3.25 | 39.3304125 |
| Feno1.13 | 39.6675625 |
| IFN100 | 0.4140573875 |
| IFN1600 | 0.0161749875 |
### Chart: HeLa DENV infection
| Category | |
|---|---|
| DMS0 | 72.05420000000001 |
| DMSO | 73.9328375 |
| Feno200 | 5.0695524999999995 |
| Feno100 | 22.8315 |
| Feno50 | 56.407475 |
| Feno25 | 72.81928750000002 |
| Feno12.5 | 78.48271249999999 |
| Feno6.25 | 78.2455875 |
| Feno3.25 | 79.3546375 |
| Feno1.13 | 75.4093625 |
| IFN100 | 21.915925 |
| IFN1600 | 2.84411625 |
### Chart: Huh751 Cell Counts
| Category | |
|---|---|
| DMS0 | 2580.625 |
| DMSO | 2765.25 |
| Feno200 | 355.5 |
| Feno100 | 830.625 |
| Feno50 | 1397.5 |
| Feno25 | 2391.875 |
| Feno12.5 | 2793.0 |
| Feno6.25 | 3085.75 |
| Feno3.25 | 2948.125 |
| Feno1.13 | 2331.125 |
| IFN100 | 1555.5 |
| IFN1600 | 1394.375 |
### Chart: HeLa Cell Counts
| Category | |
|---|---|
| DMS0 | 7784.0 |
| DMSO | 7783.125 |
| Feno200 | 1198.75 |
| Feno100 | 3441.75 |
| Feno50 | 5163.375 |
| Feno25 | 6206.5 |
| Feno12.5 | 6829.375 |
| Feno6.25 | 7226.875 |
| Feno3.25 | 7139.875 |
| Feno1.13 | 6506.75 |
| IFN100 | 4887.875 |
| IFN1600 | 4620.875 |c. d.
Figure S6. The PPARa agonist fenofibrate exhibits antiviral effect that correlates directly with high cytotoxicity. Huh7.5.1 cells (a) and HeLa cells (b) were treated with the indicated concentrations of fenofibrate or IFN followed by infection with HCV (Huh 7.5.1 cells) or DENV (HeLa cells), then incubation for 48h, followed by fixation, permeabilization, and staining, then imaging and analysis for percent infection (a,b) and total cell count (c,d).

## Slide 11
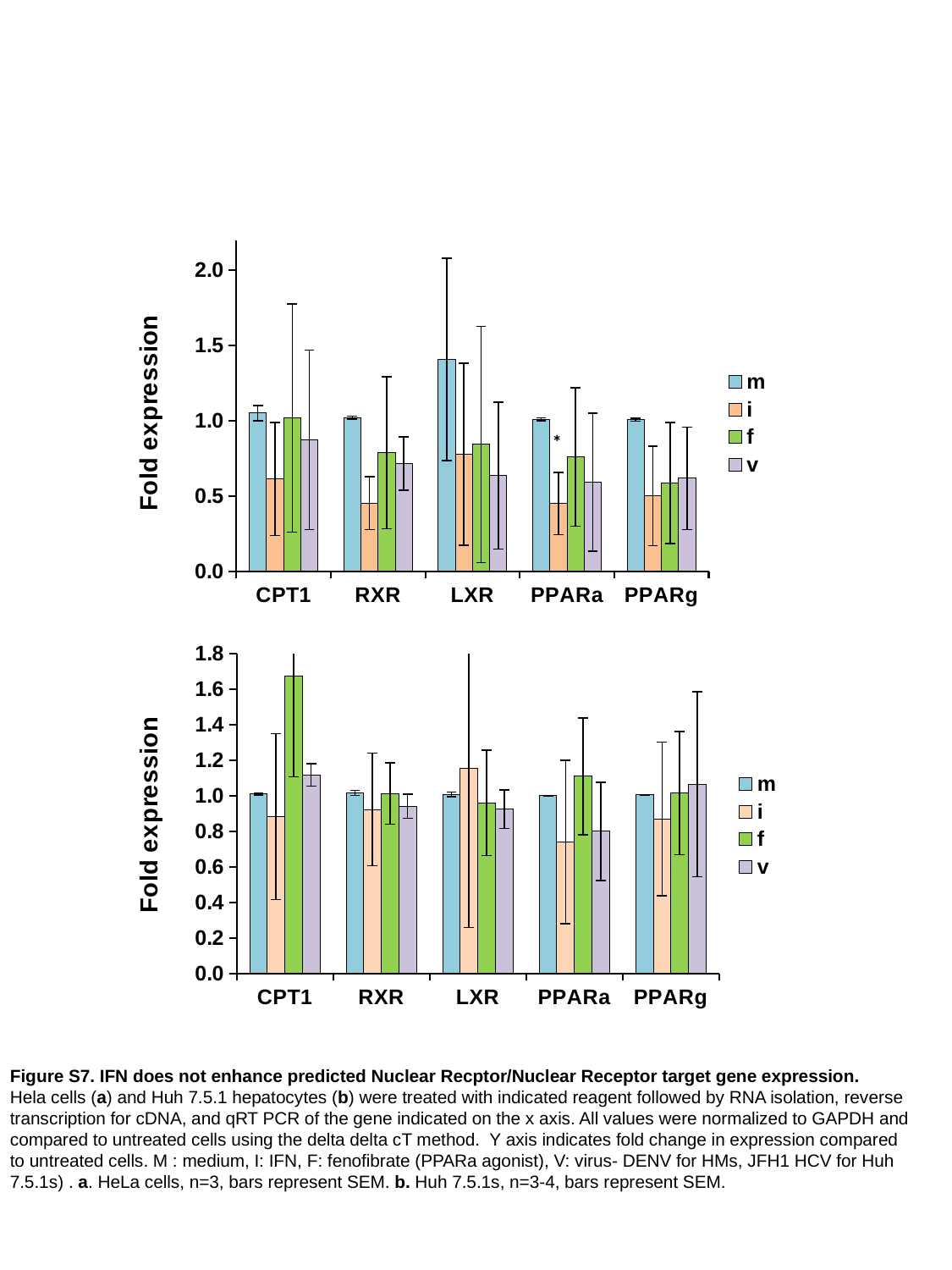

### Chart
| Category | | | | |
|---|---|---|---|---|
| CPT1 | 1.0517380874043862 | 0.611598830038001 | 1.0179449192808858 | 0.8738952726678896 |
| RXR | 1.0214466186989581 | 0.4532024519678007 | 0.7880187420890535 | 0.7156761675950971 |
| LXR | 1.4082041768219746 | 0.7771913097462932 | 0.8434248797966251 | 0.635640356591739 |
| PPARa | 1.010637027932945 | 0.44844432884619584 | 0.759207510191659 | 0.5908851843551748 |
| PPARg | 1.0080580410222726 | 0.49961592543800987 | 0.5854857974322645 | 0.6181557363825603 |
### Chart
| Category | | | | |
|---|---|---|---|---|
| CPT1 | 1.0104001319235032 | 0.8820983237315665 | 1.6750082745200425 | 1.1184513558435636 |
| RXR | 1.0163720305091533 | 0.922487100824178 | 1.0131801306570825 | 0.9408873772648322 |
| LXR | 1.0074529827511676 | 1.155816501498152 | 0.9594236185357503 | 0.9243127208853883 |
| PPARa | 1.0015040702902982 | 0.7398504415168636 | 1.1097886971500248 | 0.7993339848259363 |
| PPARg | 1.004999073876998 | 0.8699273012910487 | 1.0153509879223028 | 1.064666198544726 |*
*
Figure S7. IFN does not enhance predicted Nuclear Recptor/Nuclear Receptor target gene expression.
Hela cells (a) and Huh 7.5.1 hepatocytes (b) were treated with indicated reagent followed by RNA isolation, reverse transcription for cDNA, and qRT PCR of the gene indicated on the x axis. All values were normalized to GAPDH and compared to untreated cells using the delta delta cT method. Y axis indicates fold change in expression compared to untreated cells. M : medium, I: IFN, F: fenofibrate (PPARa agonist), V: virus- DENV for HMs, JFH1 HCV for Huh 7.5.1s) . a. HeLa cells, n=3, bars represent SEM. b. Huh 7.5.1s, n=3-4, bars represent SEM.

## Slide 12
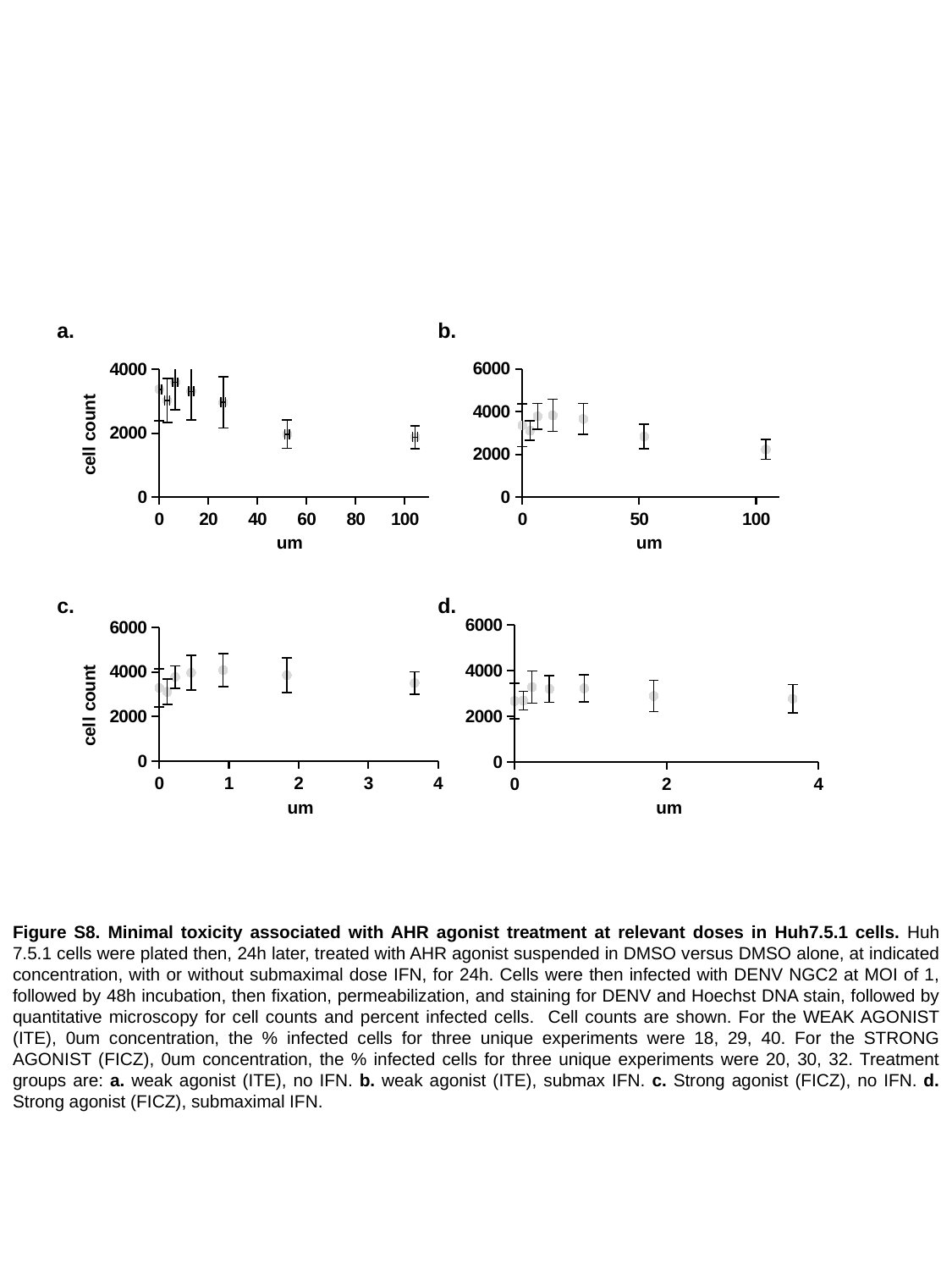

### Chart
| Category | |
|---|---|
### Chart
| Category | |
|---|---|
### Chart
| Category | |
|---|---|
### Chart
| Category | |
|---|---|a.			b.
c.			d.
Figure S8. Minimal toxicity associated with AHR agonist treatment at relevant doses in Huh7.5.1 cells. Huh 7.5.1 cells were plated then, 24h later, treated with AHR agonist suspended in DMSO versus DMSO alone, at indicated concentration, with or without submaximal dose IFN, for 24h. Cells were then infected with DENV NGC2 at MOI of 1, followed by 48h incubation, then fixation, permeabilization, and staining for DENV and Hoechst DNA stain, followed by quantitative microscopy for cell counts and percent infected cells. Cell counts are shown. For the WEAK AGONIST (ITE), 0um concentration, the % infected cells for three unique experiments were 18, 29, 40. For the STRONG AGONIST (FICZ), 0um concentration, the % infected cells for three unique experiments were 20, 30, 32. Treatment groups are: a. weak agonist (ITE), no IFN. b. weak agonist (ITE), submax IFN. c. Strong agonist (FICZ), no IFN. d. Strong agonist (FICZ), submaximal IFN.

## Slide 13
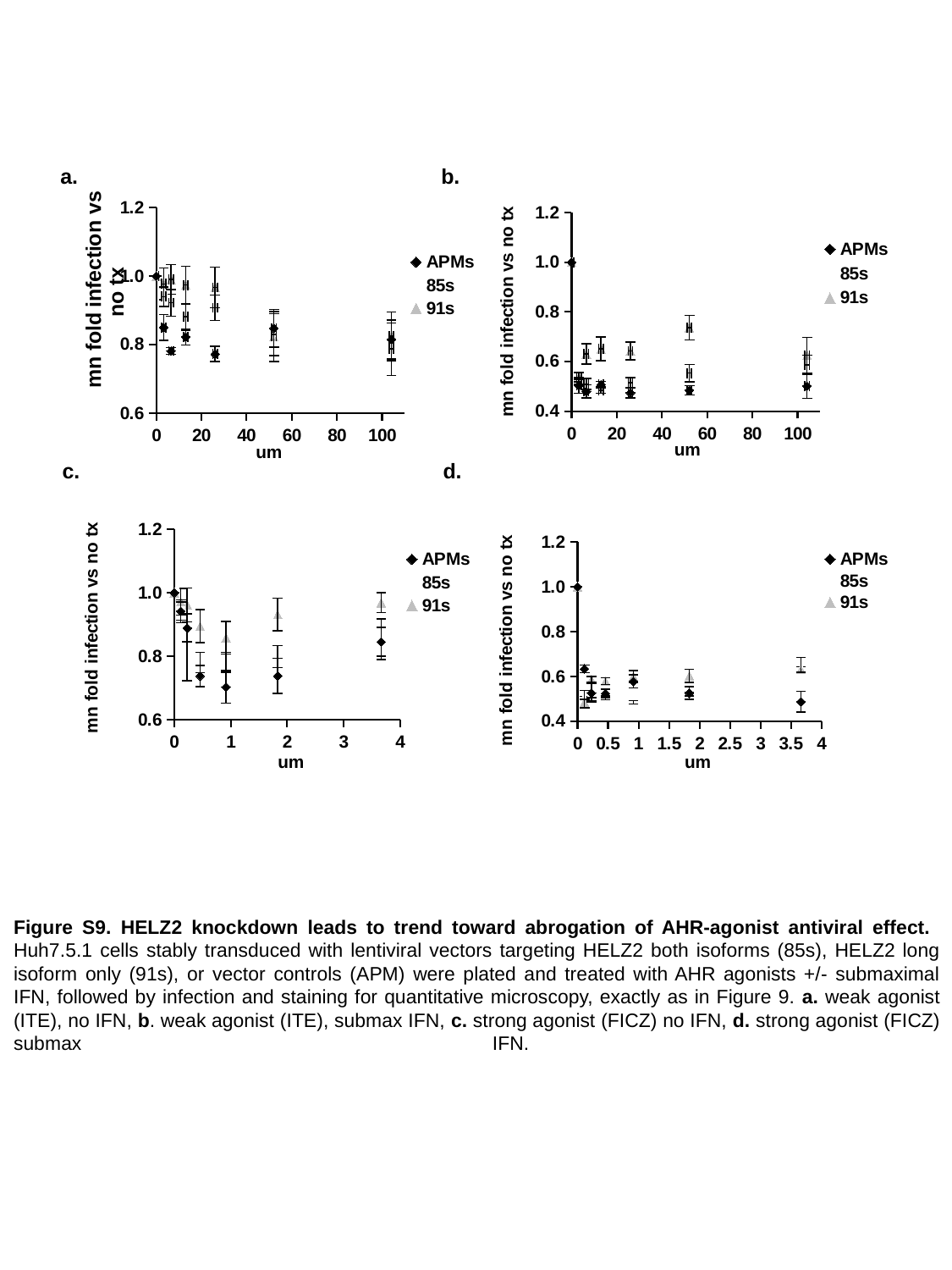

### Chart
| Category | | | |
|---|---|---|---|
### Chart
| Category | | | |
|---|---|---|---|
### Chart
| Category | | | |
|---|---|---|---|
### Chart
| Category | | | |
|---|---|---|---|
a.			b.
c.			d.
Figure S9. HELZ2 knockdown leads to trend toward abrogation of AHR-agonist antiviral effect.
Huh7.5.1 cells stably transduced with lentiviral vectors targeting HELZ2 both isoforms (85s), HELZ2 long isoform only (91s), or vector controls (APM) were plated and treated with AHR agonists +/- submaximal IFN, followed by infection and staining for quantitative microscopy, exactly as in Figure 9. a. weak agonist (ITE), no IFN, b. weak agonist (ITE), submax IFN, c. strong agonist (FICZ) no IFN, d. strong agonist (FICZ) submax IFN.

## Slide 14
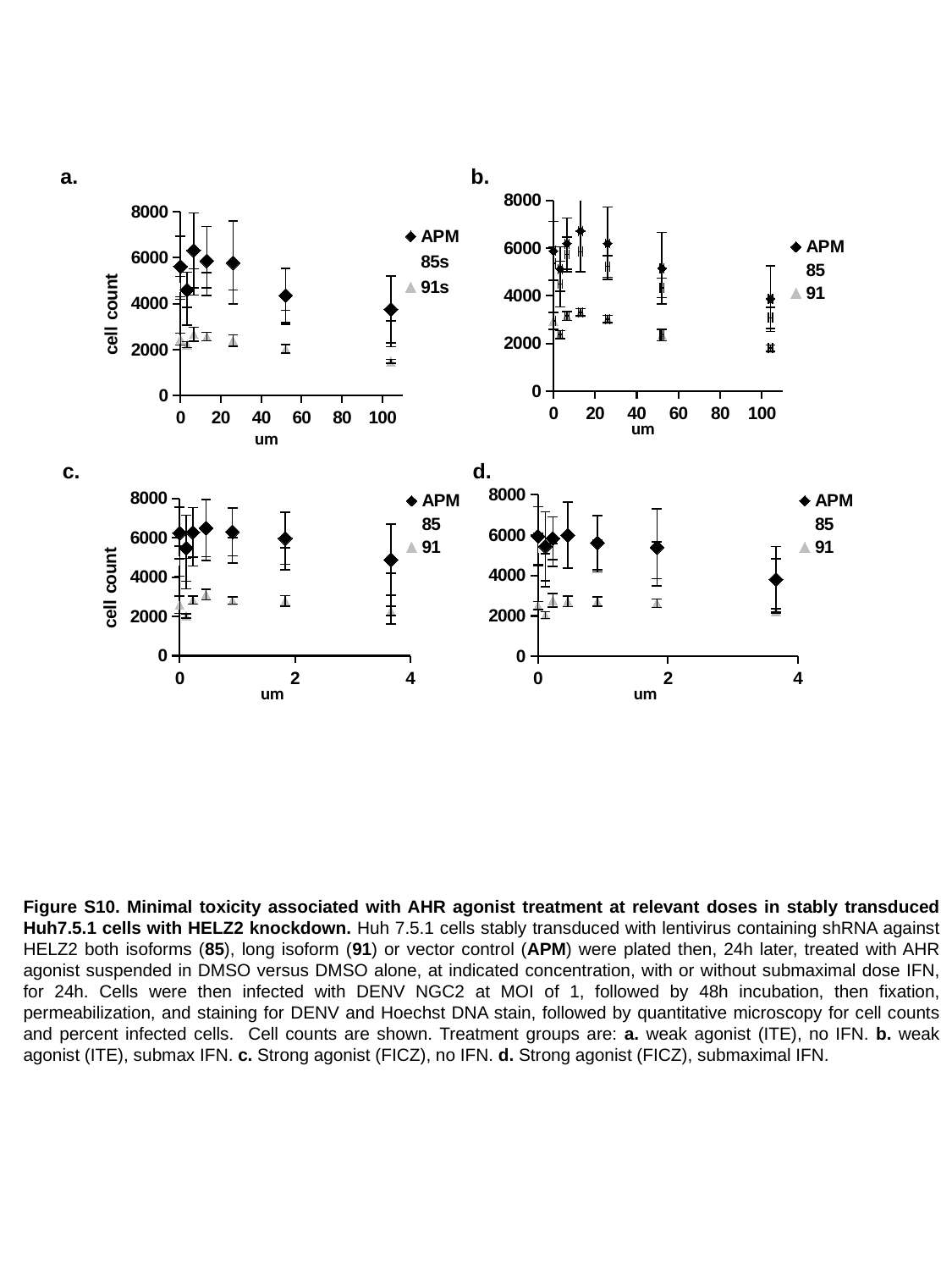

a.			 b.
### Chart
| Category | | | |
|---|---|---|---|
### Chart
| Category | | | |
|---|---|---|---|
### Chart
| Category | | | |
|---|---|---|---|
### Chart
| Category | | | |
|---|---|---|---|c.			 d.
Figure S10. Minimal toxicity associated with AHR agonist treatment at relevant doses in stably transduced Huh7.5.1 cells with HELZ2 knockdown. Huh 7.5.1 cells stably transduced with lentivirus containing shRNA against HELZ2 both isoforms (85), long isoform (91) or vector control (APM) were plated then, 24h later, treated with AHR agonist suspended in DMSO versus DMSO alone, at indicated concentration, with or without submaximal dose IFN, for 24h. Cells were then infected with DENV NGC2 at MOI of 1, followed by 48h incubation, then fixation, permeabilization, and staining for DENV and Hoechst DNA stain, followed by quantitative microscopy for cell counts and percent infected cells. Cell counts are shown. Treatment groups are: a. weak agonist (ITE), no IFN. b. weak agonist (ITE), submax IFN. c. Strong agonist (FICZ), no IFN. d. Strong agonist (FICZ), submaximal IFN.

## Slide 15
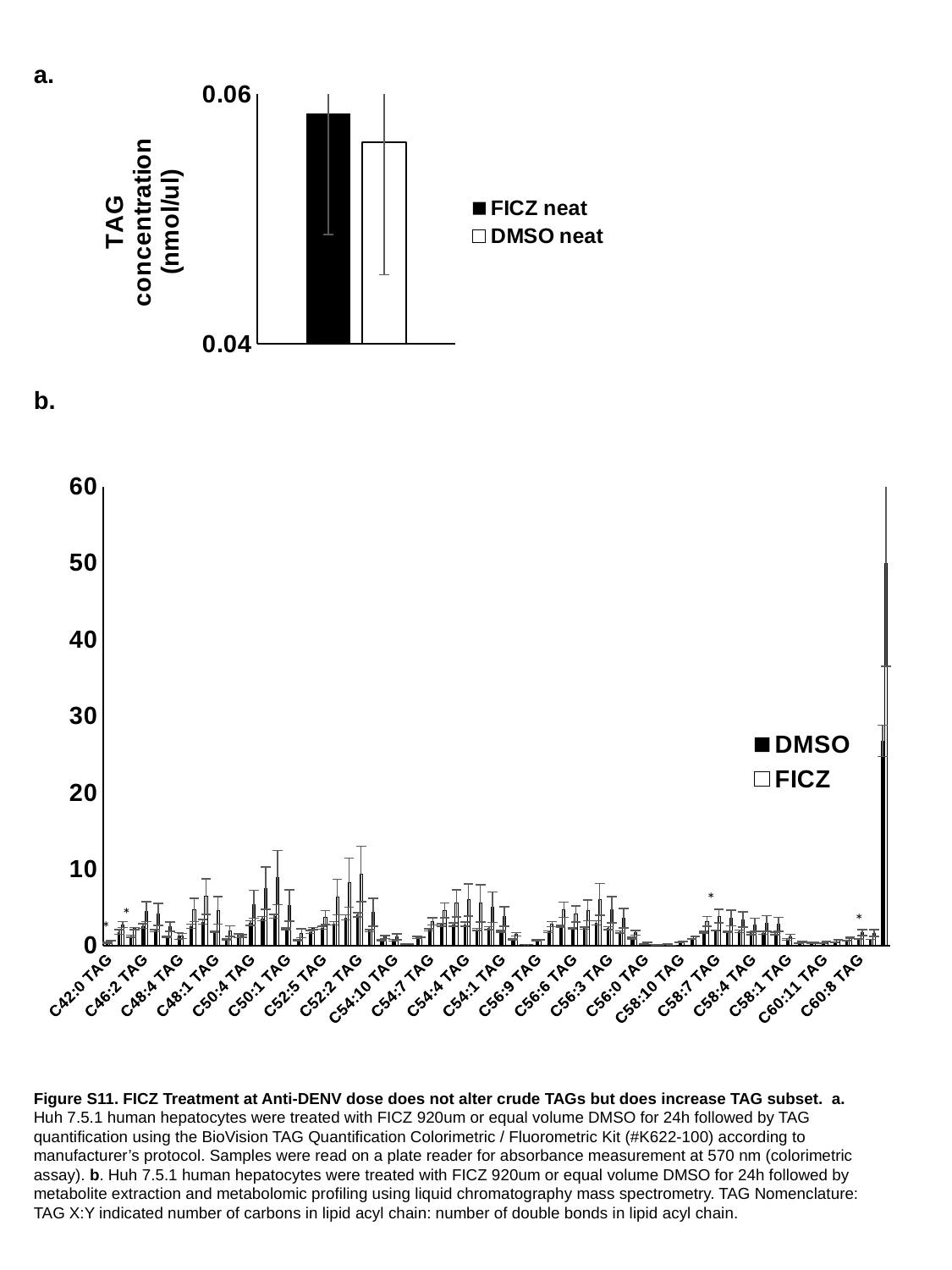

a.
b.
### Chart
| Category | | |
|---|---|---|
### Chart
| Category | | |
|---|---|---|
| C42:0 TAG | 0.3259404488661999 | 0.6182598721063363 |
| C44:1 TAG | 1.7550057672649357 | 2.7030747162455073 |
| C44:0 TAG | 1.2072279554761138 | 2.1811298704688866 |
| C46:2 TAG | 2.5104743983973457 | 4.414443470987693 |
| C46:1 TAG | 1.9025780867892201 | 4.049112918658016 |
| C46:0 TAG | 1.1089633912253787 | 2.440844272496649 |
| C48:4 TAG | 1.240177644792223 | 1.2090434252081383 |
| C48:3 TAG | 2.5075277970699186 | 4.614192913143759 |
| C48:2 TAG | 3.0661787894233683 | 6.372775231233133 |
| C48:1 TAG | 1.7708492495773662 | 4.580628431174366 |
| C48:0 TAG | 0.8118808741553939 | 1.8774236250546625 |
| C50:5 TAG | 1.2367456863344544 | 1.2156973712568688 |
| C50:4 TAG | 2.9093680229317234 | 5.369984123857642 |
| C50:3 TAG | 3.4983184199215764 | 7.469267507713164 |
| C50:2 TAG | 3.7822604003384543 | 8.852054265815251 |
| C50:1 TAG | 2.1733154454255104 | 5.198965127499942 |
| C50:0 TAG | 0.6558706683399195 | 1.5794033642579042 |
| C52:6 TAG | 1.7554470699550346 | 2.1958766256110502 |
| C52:5 TAG | 2.310644747659491 | 3.647242860536104 |
| C52:4 TAG | 2.877145511889123 | 6.310108587290845 |
| C52:3 TAG | 3.643130195488784 | 8.201589901056188 |
| C52:2 TAG | 3.9895254309579395 | 9.314886100166614 |
| C52:1 TAG | 1.9015124074936713 | 4.326737546591268 |
| C52:0 TAG | 0.6581559494578735 | 1.0692697837466185 |
| C54:10 TAG | 0.6542668724341287 | 1.1047169013498066 |
| C54:9 TAG | 0.14957941115521053 | 0.15938459830814128 |
| C54:8 TAG | 1.0297345147306247 | 1.0898511128940638 |
| C54:7 TAG | 2.0311454324470333 | 3.1075465718513557 |
| C54:6 TAG | 2.6732882419709423 | 4.577629460459258 |
| C54:5 TAG | 2.7192153851268586 | 5.487998400088712 |
| C54:4 TAG | 2.761719970467221 | 5.941647356338603 |
| C54:3 TAG | 2.08759146418605 | 5.494755306987293 |
| C54:2 TAG | 2.2374601439167034 | 4.991050010819249 |
| C54:1 TAG | 1.8359264633778363 | 3.7464458820729893 |
| C54:0 TAG | 0.8119645508658561 | 1.446559921057236 |
| C56:10 TAG | 0.10746659353841119 | 0.10230090262524819 |
| C56:9 TAG | 0.6642140548507139 | 0.7091227088379349 |
| C56:8 TAG | 1.7766326741804868 | 2.786776979140531 |
| C56:7 TAG | 2.5013764998186776 | 4.648254131324325 |
| C56:6 TAG | 2.1859565292287044 | 4.093169699112693 |
| C56:5 TAG | 2.2434875507057424 | 4.54958980699452 |
| C56:4 TAG | 2.9414535604878793 | 6.011159251795531 |
| C56:3 TAG | 2.237117303261441 | 4.6404120634519295 |
| C56:2 TAG | 1.7672131739930457 | 3.5239317906213183 |
| C56:1 TAG | 0.9334928973079787 | 1.6299195492288776 |
| C56:0 TAG | 0.135325712051646 | 0.24318964406935795 |
| C58:12 TAG | 0.055556346479929124 | 0.055404549305464616 |
| C58:11 TAG | 0.14155334184044568 | 0.0 |
| C58:10 TAG | 0.37059200046230895 | 0.45496165584777715 |
| C58:9 TAG | 0.8220506376691877 | 1.1978386527435263 |
| C58:8 TAG | 1.7175962412729457 | 3.1399698010272137 |
| C58:7 TAG | 1.9467345020035929 | 3.823457361695371 |
| C58:6 TAG | 1.7750679325403234 | 3.579472939688134 |
| C58:5 TAG | 1.8101001132368306 | 3.3700548858455956 |
| C58:4 TAG | 1.5649018935093741 | 2.695522923540162 |
| C58:3 TAG | 1.6367411387401951 | 2.853147948430907 |
| C58:2 TAG | 1.5471793461730174 | 2.7291535291354934 |
| C58:1 TAG | 0.7641312198412816 | 1.1561418277346864 |
| C58:0 TAG | 0.30089267294199046 | 0.4445576156834454 |
| C60:12 TAG | 0.3351915318605018 | 0.3844745825312383 |
| C60:11 TAG | 0.33101603451662037 | 0.46520299737425563 |
| C60:10 TAG | 0.42183370371592216 | 0.6906781544151706 |
| C60:9 TAG | 0.5646973374499307 | 0.9549622599135279 |
| C60:8 TAG | 0.8598156701025318 | 1.6764418887669013 |
| C60:7 TAG | 0.7779753760294518 | 1.6000706109715168 |
| C60:6 TAG | 26.752536648391754 | 49.94150701892042 |*
*
*
*
Figure S11. FICZ Treatment at Anti-DENV dose does not alter crude TAGs but does increase TAG subset. a. Huh 7.5.1 human hepatocytes were treated with FICZ 920um or equal volume DMSO for 24h followed by TAG quantification using the BioVision TAG Quantification Colorimetric / Fluorometric Kit (#K622-100) according to manufacturer’s protocol. Samples were read on a plate reader for absorbance measurement at 570 nm (colorimetric assay). b. Huh 7.5.1 human hepatocytes were treated with FICZ 920um or equal volume DMSO for 24h followed by metabolite extraction and metabolomic profiling using liquid chromatography mass spectrometry. TAG Nomenclature: TAG X:Y indicated number of carbons in lipid acyl chain: number of double bonds in lipid acyl chain.

## Slide 16
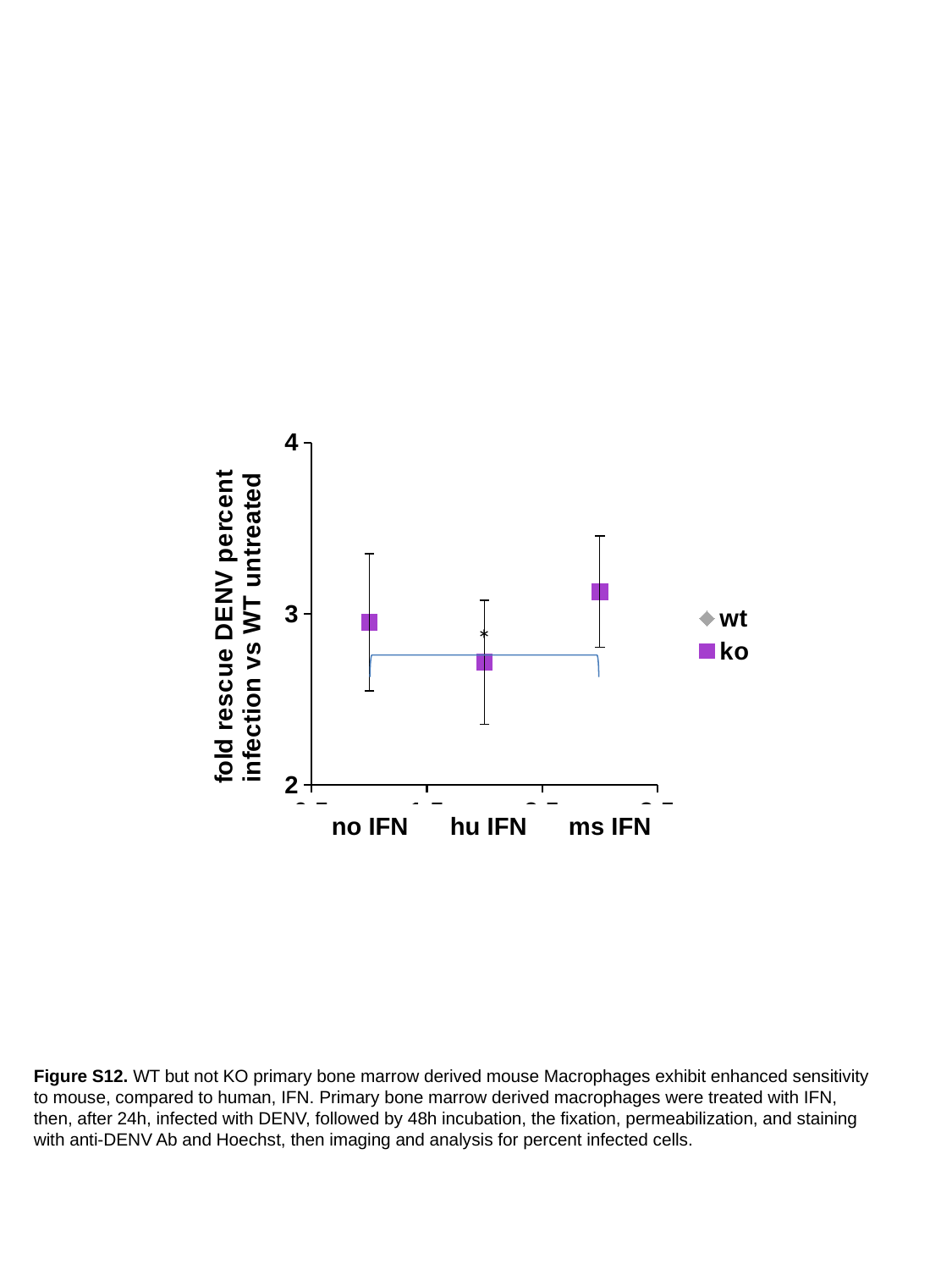

### Chart
| Category | | |
|---|---|---|
*
 no IFN hu IFN ms IFNo IFN
Figure S12. WT but not KO primary bone marrow derived mouse Macrophages exhibit enhanced sensitivity to mouse, compared to human, IFN. Primary bone marrow derived macrophages were treated with IFN, then, after 24h, infected with DENV, followed by 48h incubation, the fixation, permeabilization, and staining with anti-DENV Ab and Hoechst, then imaging and analysis for percent infected cells.
