# Supplementary material for: HELZ2 Is an IFN Effector Mediating Suppression of Dengue Virus
Source: Front Microbiol. 2017 Feb 20;8:240. doi: 10.3389/fmicb.2017.00240 (PMC5316548; doi:10.3389/fmicb.2017.00240)
Supplement: Supplementary file 3 [file DataSheet2.docx]

**Helz2-beta (negative)**

ISRE

>gi|568815578|ref|NC_000020.11|:63572170-63577384 Homo sapiens chromosome 20, GRCh38 Primary Assembly

CTGGTCGAAGGCCACCATCTGTGCGTGCTCCGAGGATGCGCAGTGGTTCTCGAAGGCCTCCTGAGAGTGG

CAGGTGACCAAGCAGGCCGGGCAGTACAGCTGGGCCCCAGGGGGCTGGCCAAGGGGGGCCGTGCGCCCGT

CGCCATCAGGGGCAGGGGGTGTGAGCAGGTCCCCCCGCTGGAGGCCACCCAGCTGCTCGGCCTCCCACAC

AGCCATCTCCACGGCGCTGTGTGCAAACTGGCAGCGGGACTCCCCACGCCAGCACGGCTGCCCACGCCCC

ACAAACCTGCAGTAGGCAGGAGGCTGGCCGGCCCGGGGCCGCGGCCTCACCACCACAAACTGTTGCTTGC

GGCGGCCCTCGGCGCTCACGTGGGCCAGGAGGGAGGGGCAGGCTCCGTGCTCAGGGCACTGCCCCTGGGG

GTCCACGCGACAGATGCATGGGGGACAGCGCCTGAAGCAGAGGGAGCAAAGCAGCTCGAAGCGGCCGCCA

AACTCCGTAAGGATGGCGTCGGCCGCCCGGCCTGCCCCTCCCTGGGCCCCGCTGCCCTGCACCTCCGCCT

TCAGCCATAGGCGCTGGAGGTTGTGCTGACGCTCGAAGGTCCAGACCAGGGCCTCCTCGCGACTGCGGGC

AAAGGTGCACCGGTTTCGGTGGCGCCGGCAGCCGAGCCCAGGGCTGTAGTAGTGGCAGACTTGGTAGCAC

AGGGGCCTTGGGAAGGTGGGCCGGCAGCCCACCACGCGCCAGACCTTGCTCTTGGTGGCCTGCTTAAAGC

GGGCCAGCAGGATCTCGCGGGAGCAGTCATGCTCCACCCTACGGAGGACGTAGGTGCTTTCATTGAGCCG

CTGGGTGCAGCGGGAGCAGCCCAGACACAGGTCCACCAGGGCACACAGCCGGGCCAGGGACGGCCCTCTG

GTGGCCGCGGGGCTGTTGGGCAGCAGGGTGGACCCTGGAGGCGCCACCTCGAACACCAGCTTGGCTCTCC

AGGGAGGCGTCTGAGGCTCCTGAGCTCACGGGTCCACGCAGTCCACTGGAACTGTTGGCCACGGTTCGAA

GCCTGCGACAGAAGGAGCAGGTGAGACTGAGGCCAGAGGACATCTGGTCCAGGCCCAGCTCAGAAGCACG

GCCTCTCCACCAGGCAAGGAGCTGGGGATCTGCCTATCCTGATGCCCAAGCAGAGGCCTCTCCACCAAGC

AAGGAGTTGGGACTCTCCCCACCCTGACGCCCAAGTGGAGGCCTTGGCAGTAGCTGTGTCCTGTCAGGGA

GTACAGGGCTCACTGACCCGGACACAGCCAGACCCTCCCTCCTGAAGGGTCTCAGGCCCCACCTAGAAGG

AGCCAAGGTGACTGCTTGTGGAGCCCTGGAGGGGGCAGAGCCCTTGGCACAGTGCCGCCCCCAGGAGGCC

GCACCCCTCTGCCCCAGCCAGGGGTCCTGTGCACCCCAGGGCCTCAGGCTCCTGACTCTTCTAAGGACAC

AGCACCTCCTTTTCTATTGAATGACCACTGGCCCCCAGGAAAAGAGGACTCCACATCTGCACCTGGGGAG

GGGCCTCACGGAGAGGAGGGTACAGTGCCCACCAGAGCTCAGGGTGCCAGGAAGAGATGGGTCCCCGGGT

CCCCACCGCAGCTGGGATGCTACAAGAAGAGTAAGTTTAAAAGCAACAGACTGGAGGCAGACTTCAGTAT

GAGAACCCGGCACTCCCCGCGGACCCCTCTGAGAAAACATCCGAGTCTGCCATCCAGAAAGGACGGGAGC

CGAGACCCCAAAATCCTGCGCTCCAGTGGGTGCAGAGACCACAGCGCTGGGTCTTGCTGCGGAGGGTGCT

GGTCCTGGACGGGCTGCACTGCCCCGGGCTGGGTCTGTGGCTCCCGGGACTCCGCGCCCCCGGCTGCACT

CACCCTCCCGGACCCCCCCCTCCGCCCCCGGCCCCGCCACCCTGGGACCCCCGCGCTCACCCTCCTGGAG

CCGCCCCTGGACCCCCGCGCCCCCGCCCCTCCGCCCTGGGACCCCCGCGCTCACCCTCCTGGAGCTGCCT

GCCCTGCCGAGCCTGCCCAGGAGCTCTCAGTTTCGATTCTGGCCCGGGCACTGACGCTGATCGCGGAAAT

TACCTCACCGCCCCCCGCCCCGCCCCGCCCTGCCCCGCCTCCAGCGACAGCTCGGCCCCCGCTGCGAGGG

AACCTCGGGAAGCGCCAGCGAGCAGAGGACGCCCCTCCCTGAAGGGCCAGCGCCGTCGAAGGCGGAGGCG

GCTGGGGGTGGGGGTCCTTTCCCGCACCCCTCCAGGGCCCCACGCCCCGTCCAGCCCTTCACACACCAGA

GACGCCCCGATGATGTGCCAGGCGGAAGGGGTGAGACCCTGCCCGGCCCTGGACGGCAGGATGGTCTGGT

GACTCCACCATCAGCCCCTGGAGAAATCCAGGCACAGCTGACCTGGAAGCCTGGGTCCCCTGACAGGCAG

GACCTGCCCGTGGCCCTGGTGCTGCTGGGACCCCAAGTCCCTTCTGTCACCAACCAGGATATGGCAGGAA

TGGCTAAGTGGCCGCCGGGTCCCAGAGTCAGCACGCCTGGCTCTTGGCTCTCTGGACTGGCTCTGGGCCC

TCAGCAGCCCTGTGGGGAGGCCCCATAGAGGAGCTGAGGCCTCCAGCCCACTTGCAGCTCGAGTCCCAGC

CCCAGCCTTACTGCCATCTCAGGGAGACCCCAGGCCTAACCCTCCCACCTAGACCACGCCCAGCTGTTAG

ATGTCTATGTTTAAAATAAACGTCCCATTAGCCAGGGGTGGGGGCGGGCGCCTGTAGTCCCAGTTACTCG

GGAGGCTGAGGCAGAAGCATTGCTTGAACCAAGGAAGTGGAGGTTGCAGTGCGCCAAGATTGCGCCACTG

CACTCCAGCCTGGGCAACAGAGCAAGACTCCATCTCAAAACAAATAAATAAATAAAATAAACATCTGTGT

TGTTTTAAGCCCCTGAGCTTTGGGGCGATCTGTTCTGCAGCAGTAGATAACGGACAGTGATGCTGCCCCT

GAAACAAGGAAGCAAGGCTGCCACCCAAGGCCAGCACCTCGTGGGGAGGGCCAGGCTGGGGGCTCACAGC

GACCTCGGGAGGTCGGCTGGGTGGGCCGTCAACCACCAGGGAGCCTCTCCGCAGACCAATCACGTTGGAG

CTCCTGGGAGAGGGCAGGCCCTCGACAATAGCAGCCCCAGGGGCCGGGCGCTGAGCAGGGTCCATGTGCG

TGGAACGCTGGGAGAATTTGTGTGGAACGCTGTGTTCCTGGATGGAGAGGCTCAACACCACACAAAGACG

TGAGTTCCTCCTAAACTAACCTGCAGTCGCATGGTGGACACACTGACTTTCAAGTCCATTTGGAAAAGCA

TCACATCACCAAGAAAGGCTGGACAGGGCTTTTTTTTTTTTTGAGACTGAGTCTCCTTCCATTGCCCCGG

CGGGAGTGCAGTGATGCAATCACAGTTCACTGCAGCCTCGACCTCTGGCTCAGACAGTCTTCCCCCCTCA

GCCTCCTGAGTCGCTGGGACTACAGGCACACACCACCACAGTACAGGTGCACACCACCACAGTTAACTAT

TTTGGAGTTTTTTCTATGTTTTTCAGAGATGGGGTTTCTCAATGTTGCCCAGGCTGCTCTTGAACTCCTG

GGCTCAAGCAAACCCCCCACCTCGGCCTCCTTGAGTGCCGGGATTAGATTACAGGCTTGAATCCCAACAC

CCAGCCCAGGATGATGACTAAAAAGGATTCCCAGCGGGTGCTGGCATGACTTACTAAACACATAACACAC

TGCAAAGCCACTGAACCCGGTGTGGACACACAGTTCCAAGGCAGAACAGAGAGTCCAGAAATAGAGTCAC

ACTCAGGAGTCTGGGGTGGGATGAAAGATTGGCTCTCGGCCAGGCGCACTGGCTCACGCCTGTAATCCCA

GCACTTTGGGAGGCCAAGGTGGGCAGATCACCTGAGGTCAGGAGTTCGAGACCAGCCTGGCCAACATAGT

GAAACCCCATCTCTTCTAAAAATACAAAAAAAAAAAAAAAAAATAGCTGGGCGTGGTGGCAGGTGCCTGT

AGTCCCAGCTACTCAGGAGGCTGAGGCAGGAGAATCCCTTGAACCTGGGGTCGGGGGGGGCGAGGCGGAG

GTTGCAGTAAGCTGAGATCACACCATTGCACTCCAGCCTGGGAGACGGAACGAGACTCTGTCTCAAAAAA

AAAAAAAAAGAAAGAAAAGAAAAGAAAAGAAAGCTTGGCTCTCACACCTCAGGGAAAAACAATTCATTGA

TGCATTCCACAAATTATTAGAACAACTGATTAGCAGCATCTGGAAACAAAAATGGGAGGGACGGTCTCTG

CGTAGCATCTTACACCATATGAAATAATCAAACACTCTCACGGAAGGAAAAGCCAACCACAGAGGCACTA

GGAGAAACCAGGGCTGAAAGTGTCTGATTCTCAGGACTCTTGGGATGTGAAAGGCCTTTCTAGGTGAAAT

CCAGCCCTGCGCCACTCGCCCCCAGCCAGGCTCAAGGACAGGGCTGGTGGCTTCACACATGCCGTGAGGG

GCAAGCATACACCCCAGAAATCCACAAAACCTGTCTCCTGAAGCACTGTTTTTTTCTTCTTAGAGAACGA

ATTAAACATCTACCAGCACAACAGTGATAAATTCAATGACACAAAACAATTTCACATGGAAAAAAACACC

ACCATCAAAGGAGTCAAAAACCAAACATGAACAGAAAACAAAATTGCAGCTTATGTCACAGTGAAAAGTC

TGATCGCTCTGATATATATTTTTTTTTCTTGAGACGGAGTCTCACTCTTGTCACCCAGGCTGGAGTGCAG

TGGCACAATCTCGGCAAGCTCCACCTCCCGGGTTCACGCCATTCTCCTGCCTCAGTCTCCCAAGTAGCTG

GGACTACAGGCGCCCACCACCACGCCTGGCTAATTTTTTGCATTTTTAGTAGAGACGGGGTTTCACCGTG

TTAGCTAGGATGGTCTCGATCTCCTGACCTCGTGATCCACCCACCTCGGCCTCCCAAAGTGCTGAGATTA

CAGGCGTGAGCCACCGCGCCTGGCCCTGATCTCTCTAATATTTTAAAGATCTTATAAATTAAGAAAAAGA

TGTATAATTTAAAAGAAAAATAGGCAAAGGATGCAAATCAATACTTCACAAAACGTGGAAAGTGAAATTG

CTGTAAATGGAGTGAAGTGTTCAACCTCGGCGTAA

V$AHR_Q5 | 20 (+) | tgtGCGTGctc

V$AHR_01 | 60 (+) | cctGAGAGTGGCAGGtga

V$AHR_Q5 | 252 (-) | cccCACGCcag

V$AHR_Q5 | 270 (-) | gccCACGCccc

V$AHR_Q5 | 342 (+) | gttGCTTGcgg

V$AHR_01 | 364 (-) | gctCACGTGGGCCAGgag

V$AHR_Q5 | 421 (-) | gtcCACGCgac

V$AHR_Q5 | 784 (+) | ctcGCGGGagc

V$AHR_01 | 977 (+) | ctcCAGGGAGGCGTCtga

V$AHR_01 | 1920 (-) | ggcCCCGCCACCCTGgga

V$ISRE_01 | 2058 (+) | cAGTTTCGATTCtgg

V$AHR_Q5 | 2278 (-) | cccCACGCccc

V$AHR_01 | 2868 (-) | ctgCACTCCAGCCTGggc

V$AHR_Q5 | 3215 (+) | tgtGCGTGgaa

V$AHR_Q5 | 3642 (-) | gctCAAGCaaa

V$AHR_Q5 | 3670 (+) | cttGAGTGccg

V$AHR_Q5 | 3902 (-) | gctCACGCctg

V$AHR_01 | 4156 (-) | ttgCACTCCAGCCTGgga

V$AHR_Q5 | 4376 (-) | tctCACGGaag

V$AHR_01 | 4809 (-) | tgtCACCCAGGCTGGagt

V$AHR_Q5 | 4862 (-) | gttCACGCcat

V$AHR_Q5 | 5041 (+) | cagGCGTGagc

V$ISRE_01 | 5166 (-) | tggAAAGTGAAATTg
