# Supplementary material for: HELZ2 Is an IFN Effector Mediating Suppression of Dengue Virus
Source: Front Microbiol. 2017 Feb 20;8:240. doi: 10.3389/fmicb.2017.00240 (PMC5316548; doi:10.3389/fmicb.2017.00240)
Supplement: Supplementary file 4 [file DataSheet3.docx]

**Supplemental Methods**

**Lifecycle Assays**

**RNA ISH Assay**

The Affymetrix protocol for ViewRNA was followed, as published (Savidis, Brass 2016). Briefly, 8mm glass coverslips were sterilized with ethanol and coated with poly-L-lysine prior to seeding HELZ2 knockdown human hepatocytes (85) or vector transduced control (APM) cells on top of the coverslips in a 24 well dish. The following day, cells were placed on ice then inoculated with DENV (m.o.i. 10) followed by a 40 minute synchronization period (allowing virus to settle uniformly on all cells) on ice. Virus was then removed followed by a 90 minute incubation in pre-warmed DMEM containing 15% FBS. Following 90 minute infection period, cells were rinsed in 1x PBS then fixed in fresh 4% paraformaldehyde, then rinsed again in PBS. The hybridization oven (GCA Precision Scientific) was set to 40˚C and given ample time to warm up prior to starting the assay. 100mm round petri dish (Fisher Cat No. 08-757-13) was labeled and lined with parafilm where the coverslips were transferred to with the seeded-cells side up. To prevent coverslips from drying out, 50µL of PBS was added on top of each of the coverslips. Affymetrix 10X detergent was diluted to 1X using PBS. The PBS on each coverslip was aspirated off with a pipette and the coverslips were incubated with the prepared detergent solution for 5 minutes at room temperature. The detergent was aspirated and the coverslips was washed twice with PBS. Affymetrix protease was diluted 1:1000 in PBS on ice and the coverslips were incubated in the prepared protease for 15 minutes at room temperature. During this time, the probe set (see probe details below) was diluted in pre-warmed (40˚C) probe set diluent. The prepared probe mix was added to the coverslips after two washes. The coverslips with the samples were then placed in the 40˚C humidified hybridization oven for 3h. Probe set PreAmp was thawed on ice and diluted 1:25 in Amplification Diluent. After 3 washes Affymetrix ViewRNA Wash Buffer, the coverslips incubated in pre-warmed 40˚C PreAMP solution for 30 minutes in the 40˚C hybridization oven. Probe set Amp was thawed on ice and diluted 1:25 in Amplification Diluent and applied for 30 minutes to the coverslips and placed in the 40˚C oven after three washes with View RNA wash buffer. Label Probe was diluted 1:25 in Label Probe Diluent and then added to the coverslips for 30 minutes in the hybridization oven. Then, the coverslips were washed three times in ViewRNA wash buffer and was left in the last wash for 10 minutes in the dark. The coverslips were mounted onto microscope slides with samples facing down using VectaShield mounting media with DAPI (Vector Laboratories H-1200). For each experiment (virus plus, probe plus), two negative controls were included (virus minus, probe plus and virus plus, probe minus).

ViewRNA Probe Set:

Dengue Virus—VF1-10726 use at 1:25

**Imaging / Analysis of Viral RNA**

Imaging of ISH experiments was performed on an inverted widefield light microscope (GE DeltaVision Elite) running softWoRx. Z stack images with 200nm spacing between slices were captured in TRITC (exposure time 300ms) followed by DAPI (exposure time 50ms) through a 60x 1.42 N.A. objective. All slides were scanned completely for positive signal in the TRITC channel. Up to 17 TRITC positive fields were collected per coverslip. When no positive signal was detected by eye, randomly selected sites were imaged to obtain control data. Stacks were deconvolved in softWoRx using the Enhanced Ratio (aggressive) method with 10 cycles. For analysis, a custom MATLAB script was written to analyze deconvolved images and measure 1) total TRITC signal per field and 2) total DAPI signal per field. Number of DAPI positive nuclei per field was also counted by hand by three independent counters. When a discrepancy was encountered, middle value for nuclei per field was selected.   Measure of virus per field were calculated by 1) total TRITC signal per field / total DAPI signal per field and 2) total TRITC per field / number of nuclei.

**qRT PCR for Dengue**

HELZ2 knockdown (85) or vector transduced control (APM) human hepatocytes were plated in 6 well dish at 300,000 cells / well-6. The following day, cells were treated with IFN 1600 iu/ml. 24h later, cells were infected with DENV at an MOI of 1. 24h later, RNA was harvested followed by reverse transcription then qRT PCR according to above protocol. DENV primers used were: GAPDH primers used were GAPDH-F: ACCTTCCCCATGGTGTCTGA, GAPDH-R: GCTCCTCCTGTTCGACAGTCA. HELZ2 primers used were HELZ2-F: GCCTGGAGTACAGCTTGAGG, HELZ2-R: CCAAGCTCCGTGTGGTATTT. DENV primers used were DENV-F: AAGGTGAGATGAAGCTGTAGTCTC, DENV-R: ATTCCATTTTCTGGCGTTCT.

**Western Blot for Dengue**

12x10^6 HELZ2 knockdown (85) or vector control (APM) human hepatocytes were plated per 10cm dish in DMEM containing 10% FBS. The following day, cells were treated with IFN 1600 iu/ml versus mock, then, 6h later, infected with DENV at an MOI of 1. The next day, protein was isolated as described above. Protein was quantified using BCA method. 30 or 40 ug of protein was then loaded (If all samples per experiment were concentrated enough to load 40 ug in 20ul, 40 ug was used for all samples. If any sample was too dilute to provide 40 ug in 20ul, 30 ug was loaded for all samples.). Due to interference between actin and dengue bands on immunoblot, identically prepared samples were run in two gels in parallel, one for actin staining for quantification, and the other for dengue staining for quantification. Following electrophoresis, transfer was performed for 90 minutes at 250 mAMPs. Following transfer, 1h block in 5% milk was performed at room temperature. After block, membranes were incubated with primary antibody diluted in 5% milk, overnight, at 4 degrees with shaking. Primary antibody dilutions were as follows: HELZ2 (abcam 129781, rabbit) 1:500, DENV 1:100 (ATCC HB-114, mouse), actin 1:10,000 (Sigma A2228, mouse). The following day, membranes were rinsed thrice for 10 minutes each in 1xTBS-T, followed by 1h incubation in appropriate HRP conjugated secondary antibody (1:5000, GE anti mouse IgG HRP NA 931V or GE anti rabbit IgG HRP NA 934V). Three more ten minute washes in 1x TBST were performed prior to development. ThermoScientific SuperSignal chemiluminescent substrate was required to visualize HELZ2 and DENV. For actin, standard ThermoScientific ECL substrate was used. Membranes were allowed to sit in their respective substrate mixtures for five minutes before development. Proteins were visualized on blue X-Ray films; for each membrane, exposures of 2s, 5s, 10s, 30s and 2min were performed. When bands appeared were too dark for quantitative analysis even after 2 seconds, repeat exposures were performed after allowing the membranes to sit in the dark room for several minutes to allow luminescence to fade.

**Supernatant Transfer Assay for Plaques**

HELZ2 knockdown (85) or vector transduced control (APM) human hepatocytes were plated in 6 well dish at 300,000 cells / well-6. The following day, cells were treated with IFN 1600 iu/ml. 24h later, cells were infected with DENV at an MOI of 1. 24h later, supernatant was collected for supernatant plaque assay on Vero cells, according to published methods. Briefly, on Day 3 post infection, supernatant from DENV infected 85 and APM cells was collected in falcon tubes 15ml. Supernatant was then aspirated from Vero cells plated 24h before on 6-well plates at 2x10^6 cells/well, and 200uL of the 85 or APM cells was applied to different wells. The 6-well Vero plates were incubated for 1 hour in 37C incubator (including gentle shaking every 15 minutes – adsorption period), and at the end of 1 hour, overlay media including agar was applied on top of the infected Vero wells [ A)Liquid phase overlay: ( i)Opti-MEM [(Temin's modification) (2X), no phenol red, Thermo Fisher Scientific, cat.# 11935046] 15ml/6-well plate, ii)FBS (Fetal Bovine Serum, Qualified, USDA-approved Regions, Fisher Scientific Gibco, product # 10437028) 1.5ml/6-well plate, iii)Ultrapure water (UltraPure™ DNase/RNase-Free Distilled Water, Thermo Fisher Scientific, cat.# 10977023) 7.5ml/6-well plate) PLUS B) Solid phase overlay: 1.5% agarose SeaKem LE AGAROSE 125G, Lonza, product # 50000 heated in microwave oven for 3 minutes]. The plates were left on RT for 15min so that agar solidified, and then were placed in 37C incubator. On day 7 (or day 4 post-supernatant infection), the plates were fixed (3ml of 10% formaldeheyde/well for 30min added directly on top of the wells), then formaldeheyde was aspirated, the solidified agar overlay was removed mechanically (reverse plate on a tray and help agar fall off without touching the well bottom), and 1 ml of 0.05% of Crystal violet dye (1ml of 2.3% Crystal violet solution, 10% ethanol, Sigma Aldrich, product # HT90132-1L in 49ml of Distilled water) was added for 30min. Then, wells were washed under running sink water and plaques were counted.

**TAG Assay for FICZ versus DMSO treated cells**

Huh 7.5.1 human hepatocytes were treated with FICZ or DMSO for 24h followed by TAG quantification using the BioVision TAG Quantification Colorimetric / Fluorometric Kit (#K622-100) according to manufacturer’s protocol. Samples were read on a plate reader for absorbance measurement at 570 nm (colorimetric assay).

**Metabolite Extraction and Metabolomic Profiling for FICZ versus DMSO treated cells**

Protocol was performed as above (for original HELZ2 knockdown samples) with the following adaptations. On initial run, there was no difference detected between samples, with very high signals in all samples, raising concern for failure of differential detection due to signal saturation. All samples were thus diluted and re-run. During re-run, one treated sample was not injected properly, leading to 3 DMSO and 2 FICZ analyzable samples. A normalization control was included, spiking the same amount internal standard (a lipid non existent in the human species, C17:1 LPC) in extraction buffer, followed by use of the same volume of this extraction buffer to treat each sample then loading of 10ul of these treated samples onto the MS to detect the lipid signal. Theoretically the internal standard signal should be identical in all the samples, but due to the unknown reason (such as the minor sample loading problems, wired chromatography issues etc.), the detected signals in different samples are always slightly different, so all the endogenous lipid species are normalized to one of the spiked internal standards in the same sample to correct the errors.
